# Risk Factors and Preventing Strategies of Pocket Hematoma After Cardiac Implantable Electronic Device Implantation: A Systematic Review

**DOI:** 10.3390/jcdd12120490

**Published:** 2025-12-12

**Authors:** Siyin Ding, Xiaohong Pan

**Affiliations:** 1 Department of Cardiology, The Second Affiliated Hospital, School of Medicine, Zhejiang University, Hangzhou 310009, China; 2317062@zju.edu.cn; 2 State Key Laboratory of Transvascular Implantation Devices, Hangzhou 310009, China; 3 Heart Regeneration and Repair Key Laboratory of Zhejiang Province, Hangzhou 310009, China

**Keywords:** pocket hematoma, cardiac implantable electronic devices, antithrombotic therapy, perioperative management, complication prevention

## Abstract

Pocket hematoma is a common complication following cardiac implantable electronic device (CIED) implantation, traditionally perceived as a manageable local issue. Accumulating evidence, however, indicates that clinically significant pocket hematoma (CSH) is strongly associated with increased infection rates, elevated healthcare costs, and heightened mortality. Key risk factors include advanced age, low body mass index (BMI), chronic kidney disease, complex procedures (device upgrades/replacements) and periprocedural antithrombotic management, particularly uninterrupted dual antiplatelet therapy (DAPT) and heparin/low-molecular-weight heparin (LMWH) bridging strategies, which significantly elevate bleeding risk compared to continued vitamin K antagonist (VKA) therapy or direct oral anticoagulant (DOAC) protocols. Novel compression devices and topical hemostatic agents show promise for prevention, while standardized definitions and risk stratification tools are urgently needed. This review synthesizes current evidence on multifactorial pathogenesis, adverse outcomes, and evolving preventive strategies for pocket hematoma, emphasizing its underappreciated clinical significance and the critical need for optimized periprocedural management in high-risk patients.

## 1. Introduction

Cardiac implantable electronic devices (CIEDs), including pacemakers, implantable cardioverter–defibrillators (ICDs), and cardiac resynchronization therapy (CRT) devices, effectively improve quality of life and prolong survival in selected patients. As CIEDs implantation rates and device complexity increase globally [1], postoperative complications such as pocket hematoma have gained clinical attention.

### 1.1. Definition of Pocket Hematoma

Pocket hematoma is typically defined as palpable welling beyond the range of the pulse generator, maybe accompanied by skin ecchymosis, local pain and increased pressure or even fluctuation within the pocket by palpation [2]. While most cases resolve spontaneously, approximately 10% require additional intervention such as blood transfusion, surgical drainage, prolonged hospitalization or readmission, interruption of anticoagulation therapy [3], which are termed “severe pocket hematoma” or “clinically significant hematomas (CSH)” and are commonly used as endpoints in clinical studies [4,5,6,7].

### 1.2. Incidence

Pocket hematoma is the most frequent complication following CIED implantation [8]. However, reported incidence varies significantly in the cited literature, primarily due to differences in hematoma definitions, followed by heterogeneity in study design, patient characteristics, antithrombotic regimens, and device types. The overall incidence of hematoma events has values which typically range between 2.0% and 5.0%, based on recent cohorts [9,10,11,12,13]. Some studies report higher values of up to 9.3% [14], often influenced by patient demographics or procedural factors. The incidence of hematomas requiring intervention is approximately 1.0% to 2.0% [9,10,15], as only a subset of hematomas necessitate invasive management due to symptoms like pain, expansion, or infection risk.

### 1.3. Pathophysiologic Mechanism

The pathophysiology of pocket hematoma involves a complex interplay of key factors. The initiating factor is microvasculature disruption within the pocket induced by the procedural trauma. In patients with advanced age, chronic kidney disease, and comorbidities like heart failure, underlying microvascular fragility may exacerbate this initial injury. Afterward, the presence of anticoagulant action critically influences hematoma risk. In particular, the postoperative administration of low-molecular-weight heparin (LMWH) poses a high risk due to its rapid onset and potent peak effect, which impair clot formation at the surgical site [16]. Concurrently, the confined space of the device pocket means that any ongoing bleeding increases intra-pocket pressure and may rapidly compromise tissue perfusion and viability, as evidenced by the limited efficacy of vacuum drainage systems [17]. Therefore, the prevention of pocket hematoma necessitates a multifaceted approach, involving both the mitigation of risk factors and the timely implementation of necessary prophylactic measures.

### 1.4. Adverse Effect

CSH is associated with multiple adverse outcomes, including infection risk [18,19,20], anticoagulation interruption [4,21], reoperation [9,22], perioperative pain, impaired quality of life [4], prolonged hospitalization [11,12,23,24,25,26,27] and elevated healthcare costs [11]. Pocket infection is a major concern since it can be life-threatening or a source of undue morbidity [13]. Meta-analyses show that CSH increases the odds of infection by eightfold [18], with reoperation raising this risk 15-fold [28]. Notably, data from the WRAP-IT trial demonstrated that hematoma was associated with an 11.3-fold increased risk of major CIED infection (HR: 11.3; 95% CI: 5.5–23.2; *p* < 0.001) [29]. Hematoma or bleeding complications add USD6,995 to the admission cost of ICD implantation and extend stays by three days [30]. When infection occurs, mortality increases 4.4–7.7-fold, with additional costs exceeding USD14,360–16,498, depending on the type of CIED [31]. Thus, hematoma prevention was emphasized to be the most important intervention in the prevention of CIED infection, besides preoperative antibiotic administration [32].

### 1.5. Grade

Pocket hematoma severity is often graded as follows [33]: Grade 1 involves an ecchymosis or a mild effusion without intervention; Grade 2 includes remarkable and symptomatic effusions needing minor treatment (not included in Grade 3); and Grade 3 comprises cases requiring reoperation, hospitalization extension, or anticoagulation reversal. Hemoglobin drops may also serve as an adjunct marker [34].

### 1.6. Measurement of Pocket Hematoma

Aspiration yields diagnostic proof if blood can be drawn by a sterile syringe or surgical drainage from the pocket, yet this is rarely performed. Clinicians therefore rely on (1) imaging: since the magnetic resonance imaging (MRI) is relatively contraindicated in a majority of CIEDs, and standard CT suffers from lead artifacts, ultrasound is the first-line tool [35]; dual-source CT with metal-artifact reduction is an emerging alternative. (2) Physical examination: palpable mass or ecchymosis is recorded, and a surface area >100 cm [2] is often classified as large [36]; this method is simple but operator-dependent. (3) Indirect grading by clinical consequences (transfusion, drainage, prolonged stay), as outlined previously. Objective and reproducible quantification remains an unmet need.

### 1.7. Time of Formation

Most pocket hematomas occurred within 1 week post-CIED procedure, with a mean time of 5 days [3,4,9,10,23,24,27,37,38]. Notably, the ESS-PREDI study reported that most of the severe pocket hematoma occurred following discharge from hospital, and the majority of minor pocket hematoma occurred during hospitalization [38].

## 2. Methods

### 2.1. Search Strategy

The Preferred Reporting Items for Systematic Reviews and Meta-Analyses (PRISMA) protocol for systematic review guidance was followed [39]. The completed PRISMA checklist is available in Table S4. A comprehensive literature search was performed in PubMed and EMBASE from January 1995 until June 2025. Key search terms included “pacemaker”, “cardiac implantable electronic device”, “CIED”, “implantable cardioverter-defibrillator”, “cardiac resynchronization therapy”, “cardiac rhythm device” in combination with “pocket hematoma”, “hematoma”, “bleeding complication” or “anticoagulant”, “antiplatelet”, “dual antiplatelet therapy”, “DAPT”, “vitamin K antagonist”, “warfarin”, “direct oral anticoagulant”, “DOAC”. These terms were combined using Boolean operators (AND, OR). Only full articles in English were included. Reference lists of relevant reviews and included studies were manually screened to identify additional records.

### 2.2. Eligibility Criteria

Studies were included if they met the following criteria: (1) original research articles, including randomized controlled trials (RCTs), prospective or retrospective cohort studies, case–control studies, or case series with a sample size of at least 10 patients; (2) participants were adult patients (aged ≥18 years) undergoing CIED implantation (e.g., pacemakers, ICDs, or CRT devices); (3) studies reported on the incidence, risk factors, prevention, management, or outcomes of pocket hematoma or bleeding complication; (4) studies were published in English. Exclusion criteria were (1) non-original articles such as reviews, editorials, commentaries, or case reports (fewer than 10 patients); (2) studies that did not mention CIED-related pocket hematoma (e.g., those involving other complications or devices); (3) studies with insufficient data or overlapping populations; (4) non-English publications to avoid translation biases.

### 2.3. Study Selection and Data Extraction

**Figure 1:**
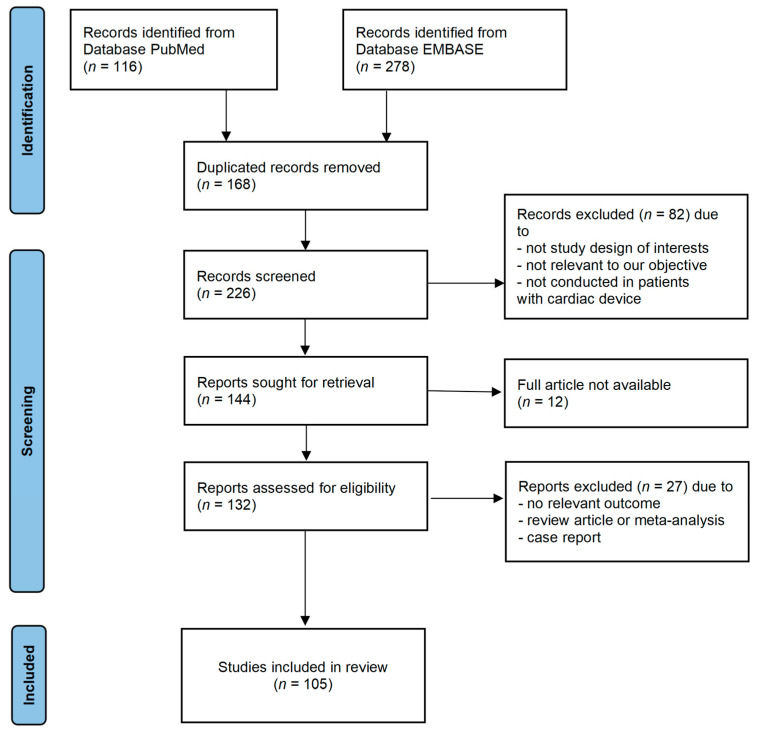
PRISMA flowchart of included articles.

Initially, all identified records were imported into EndNote X9 (Clarivate Analytics, Philadelphia, PA, USA) to remove duplicates. Two independent reviewers screened titles and abstracts for eligibility based on the inclusion criteria. Full-text articles will be assessed by the same reviewers, with disagreements resolved through discussion. The study selection process is summarized in the PRISMA flowchart (Figure 1). A standardized data extraction form was used to collect information from the included studies. Extracted data included first author, publication year, study design, patient population characteristics, number of participants, CIED type, details of periprocedural antithrombotic management (drug, timing, interruption/bridging protocols), incidence of overall pocket hematoma and CSH, identified risk factors, and reported preventive or treatment strategies. A narrative synthesis was conducted due to the heterogeneity in study designs, populations, and outcomes. Studies were grouped by themes (e.g., risk factors such as antithrombotic therapy, and preventive strategies such as surgical techniques) based on the extracted data. Key findings on were summarized using structured tables (Table S1 see Supplementary Materials) and textual descriptions to facilitate comparison and theme identification.

### 2.4. Quality Assessment

The quality of included randomized controlled trials was assessed using the Cochrane Risk of Bias tool 2. Observational studies were evaluated using the Newcastle–Ottawa Scale (NOS) for assessing the quality of non-randomized studies.

The systematic review was registered with PROSPERO (international prospective register of systematic reviews), registration number CRD420251208331.

## 3. Risk Factors of Pocket Hematoma

Identifying risk factors enables targeted prevention, particularly in patients receiving antithrombotic therapy. Ongoing debates underscore the need for updated evidence.

### 3.1. Patient Characteristics

#### 3.1.1. Advanced Age

Advanced age is remarkably linked to hematoma formation and attributed to lax subcutaneous tissue and poorer muscle tone [40]. Large-cohort studies and the SIMPLE trial confirm age as an independent predictor [41,42]. Yet, some studies report higher rates in younger patients, likely reflecting comorbidity and antiplatelet use variability [10,43]. It was pointed out that the younger patients who need pacemakers may have more complications than older patients due to degeneration of the conduction pathway.

#### 3.1.2. Sex

Female sex may confer modestly increased risk [44], plausibly mediated by lower BMI and smaller vessel diameter [45], although data remain inconsistent.

#### 3.1.3. Low Body Mass Index (BMI)

Low BMI consistently increases hematoma risk [46,47], while obesity or being overweight seems to be a protective factor against pocket hematoma [27,48]. Thin subcutaneous tissue offers limited tamponade, whereas obesity masks swelling and redistributes blood, lowering clinically evident hematomas. According to the ESS-PREDI study, the underweight patients (BMI < 18 kg/m^2^) had a higher rate of minor pocket hematoma (31.3%) compared with normal weight patients (BMI 18 to < 25 kg/m^2^; 8.3%), overweight patients (BMI 25 to < 35 kg/m^2^; 7.1%), or morbidly obese patients (BMI ≥ 35 kg/m^2^; 10.8%) [38]. Guo et al. [49] reported an increased incidence of pocket hematoma in patients with BMI < 23 kg/m^2^, presumably due to the thinner subthoracic fat. Consequently, it is suggested that subpectoral implants and avoiding heparin bridging in patients with BMI < kg/m^2^ [50].

### 3.2. Comorbidities

#### 3.2.1. Chronic Kidney Disease (CKD)

CKD is an established independent predictor [5,7,27,40], and risk rises progressively with declining GFR [5,48]. Uremic platelet dysfunction and delayed clearance of antiplatelet or anticoagulant drugs underlie this association.

#### 3.2.2. Congestive Heart Failure

In early studies, neither heart failure nor left ventricular ejection fraction (LVEF) was associated with an increased risk of pocket hematoma [23,26]. However, heart failure, especially LVEF below 30%, has emerged as a potent predictor in recent studies [34,40], largely mediated by concomitant anticoagulation for comorbidities such as atrial fibrillation [40].

#### 3.2.3. Hypersensitivity Reaction

Rare hypersensitivity reactions to device components may manifest as recurrent pocket hematoma [51]. Bai et al. reported a case of a 64-year-old female patient repeatedly suffering pocket hematoma following permanent pacemaker implantation [47]. The histopathological examination of the pocket exhibited the proliferation of granulation tissue with multinucleated giant cells with negative bacterial culture, and the hematoma was absent following treatment with promethazine for one week. Further analysis proved the efficacy of promethazine treatment in significantly reducing the incidence of reoperations and the duration of hospital stay in nine patients with medical histories of allergic reactions who were suffering from pocket hematoma [47]. Results demonstrated that the rate of pocket hematoma was significantly higher in patients with a history of allergies when compared with those without a history of allergies [47]. Because such reactions are uncommon and mimic infection, larger studies are needed to confirm allergy as an independent risk factor.

#### 3.2.4. Thrombocytopenia

Moderate-to-severe thrombocytopenia increases hematoma risk fourfold [52]. Demir et al. declared that baseline platelet level (β = −0.027, OR = 0.974, 95%CI = 0.951–0.997, *p* = 0.025) was one of the independent risk factors for pocket hematoma formation [53]. Current guidelines offer no specific thresholds; therefore, withholding or reducing antithrombotic agents and considering perioperative platelet transfusion should be individualized to minimize bleeding.

### 3.3. Implantation-Related Factors

#### 3.3.1. Type of Device

High-complexity devices such as CRT-D and dual-chamber ICDs are linked to increased CSH risk due to larger generator size, multiple leads, and longer procedure duration [11,15,26,34,47,54,55,56,57,58]. Notably, the indication for a more complex CIED operation is likely a more severe underlying heart disease, which places these patients at a higher risk of pocket hematoma. However, some studies found no significant difference [25,40,42,48,59,60], possibly due to operator experience bias, as complex procedures are often performed by senior physicians [2,9,25].

#### 3.3.2. Replacement/Upgrade

Device replacements or upgrades, particularly those involving lead additions or repositioning, carry higher hematoma risk due to fibrotic tissue dissection and prolonged operative time [9,15,42,48,61,62]. Reoperations are consistently associated with increased bleeding complications compared with de novo implants.

#### 3.3.3. Position of Pocket

Submuscular placement, once favored to prevent erosion, is now associated with higher hematoma rates due to transection of the pectoralis major muscle, accompanied by inherent bleeding risk [9,42,63]. With modern smaller devices, subcutaneous implantation is increasingly preferred, particularly in thin patients with limited tissue coverage [9,64].

#### 3.3.4. Technique of Implantation/Operator Volume

Operator inexperience is a recognized risk factor [9]. Hematoma rates are significantly higher when procedures are performed by trainees or low-volume operators [25,58]. In patients on dual antiplatelet therapy which cannot be discontinued, implantation should ideally be performed by experienced physicians to minimize complications [9].

#### 3.3.5. Duration of Procedure

Prolonged procedure time has been associated with increased hematoma risk, particularly in anticoagulated patients [65,66]. However, its independence as a predictor varies across studies, likely due to confounding factors such as device complexity and antithrombotic use [27].

#### 3.3.6. Venous Approach

Early data suggested higher hematoma rates with subclavian puncture [9,26] due to difficulty in achieving hemostasis [67], while cephalic cutdown allows direct visualization and controlled lead placement into the vessel should minimize late back-bleeding into the pocket [26]. However, recent studies and meta-analyses report no significant difference between routes [60,66,68,69,70,71]. Specifically, axillary vein access guided by fluoroscopy or ultrasound is associated with higher procedural success rates, shorter procedure times, and comparable safety profiles regarding hematoma, pneumothorax, and lead failure [71,72,73,74].

### 3.4. Antithrombotic Therapy

Growing proportion of CIED candidates receive antiplatelet or anticoagulant therapy for cardiovascular or cerebrovascular protection. Aging demographics and broader guideline indications have expanded the spectrum of perioperative antithrombotic combinations. Major published studies regarding the pocket hematoma incidence post-CIED procedures in the setting of antithrombotic therapy and the bleeding complication rate are summarized in Table S1 and Table S2, respectively.

#### 3.4.1. Antiplatelet Therapy

Up to 40–58% of contemporary CIED recipients are on antiplatelet therapy [27,75,76], most commonly acetylsalicylic acid (ASA), thienopyridines (ticlopidine/clopidogrel), and ticagrelor, reflecting the high burden of coronary artery disease. Regardless of anticoagulant status, antiplatelet use was associated with a doubling of risk compared to no antiplatelet group [77].

##### Single Antiplatelet Therapy (SAPT)

Single antiplatelet therapy (SAPT) with aspirin or clopidogrel marginally raises bleeding tendency [26,66,67], yet does not significantly augment clinically relevant hematoma rates [4,9,10,27,37,65]; therefore, routine discontinuation is not warranted. Notably, aspirin is generally considered safer than other antiplatelet agents (e.g., clopidogrel) in terms of hematoma risk, as evidenced by studies showing no significant increase in bleeding complications with aspirin monotherapy compared to no therapy, while clopidogrel alone may pose a higher risk [9,12,78]. Meticulous intra-operative hemostasis appears more important than drug interruption [12].

##### Dual Antiplatelet Therapy (DAPT)

The percentage of patients on uninterrupted DAPT at the time of CIED implantation was about 8.5% [12,60], and has developed progressively with the annually increasing use of stents in coronary interventions [69]. Uninterrupted dual antiplatelet therapy (DAPT) is one of the strongest pharmacological risk factors for CSH, with bleeding rates of 13.3% to 24.5% [9,12,27,38,52,53,58,60,65,75] roughly twofold that of SAPT [65], and 5–10 times higher than with no therapy [9]. Meta-analysis of 5978 patients confirmed an adjusted odds ratio of 5.0 for DAPT-related bleeding [37]. It was reported that enhanced ADP-mediated platelet inhibition, rather than aspirin effect, correlates with hematoma occurrence [79]. In light of this, if possible, it is recommended to discontinue one antiplatelet agent (typically the P2Y12 inhibitor) for 5–7 days before CIED implantation to reduce bleeding risk, especially in elective procedures, while carefully assessing the thrombotic risk based on individual patient factors [26,65,78,80].

However, a few studies insisted that pocket hematoma risk does not seem to increase with DAPT agents [10,60,69,79,81], in spite of limited sample size. Dreger et al. reported an extremely low and comparable incidence of pocket hematoma in a cohort of 109 patients with DAPT and 318 controls with single or no AP treatment, but in this study, a vacuum drainage system was applied to all patients [69].

#### 3.4.2. Anticoagulant Therapy

Almost 22.9–37.9% of patients are receiving PPMs, and 17.5–37% of those receiving ICDs are taking OAC alongside the procedure [3,4,27,61,76,82,83].

##### Vitamin K Antagonist (VKA)

Continued warfarin with INR above 2.5 may modestly increase bleeding [3,84,85], but with INR in the lower therapeutic range (2.0–2.5), this does not raise CSH rates above interrupted strategies and is safer than bridging [4,22,36,54,75,85,86,87,88,89,90,91]. The BRUISE CONTROL-1 trial demonstrated an 80% reduction in pocket hematoma with continued warfarin (3.5%) versus heparin bridging (16.0%) [4]. Consequently, uninterrupted warfarin is preferred for patients with moderate-to-high thromboembolic risk, and the INR target is ≤2.5 and can be relaxed to ≤3.0 for patients with mechanical valvular heart disease [78].

##### Heparin/Low Molecular Weight Heparin (LMWH)

Heparin bridging is substantially associated with a significantly higher incidence of hematoma, ranging from 5.7% to 25% [2,4,9,21,23,24,26,37,52,54,68,77,85,88,89,92,93,94,95], and is identified as a strong risk factor for CSH formation [48,79,96]. Hematomas often present later because summative anticoagulant effect from resumed warfarin and postoperative LMWH peaks at day 3–4 [27]. Randomized data indicate that preoperative LMWH is not harmful, whereas any postoperative dose increases hematoma rates [21]. The heightened vulnerability is explained by rapid, high-level anticoagulation that hampers early wound sealing, contrasting with the stable, moderate effect of therapeutic warfarin [97]. Another explanation is the potent inhibition of more elements of the coagulation cascade and the prevention of a clot/thrombus from the synergistic action of heparin/LMWH [79]. Aside from that, Birnie et al. [16] supported the concept of “anticoagulant stress test” [21], that is, in the setting of operatively continued warfarin, bleeding is more likely to be detectable and managed while the wound is still open so as to avoid the following spontaneous development of pocket hematomas, while bleeding after wound closure that may be exacerbated by postoperative heparin is insidious and not easily managed.

Multiple meta-analyses suggested fewer bleeding complications and similar risks of thromboembolism with uninterrupted OAC as compared to heparin bridging [37,56,91,98,99,100,101,102]. Consequently, current guidance discourages routine bridging and favors uninterrupted oral anticoagulation.

##### Direct Oral Anticoagulants (DOACs)

Early studies described the rate of bleeding complications following CIED implantations of patients treated with dabigatran as extremely varied, ranging from 2% to 30% [3,103,104,105,106,107,108], while that of rivaroxaban was reported from 5% to 55.6% [3,104], probably due to the considerable provider variability in DOAC management prior to CIED procedures [109]. A large observational study identified continued DOAC as an independent predictor of major bleeding (pocket hematoma 9.8%, CSH 4.5%), whereas interruption caused thromboembolic deaths [110]. However, plenty of studies demonstrated that the security in bleeding complications of periprocedurally continued DOACs [16,38,105,111,112,113,114,115]. A meta-analysis of three studies (773 subjects) showed no significant difference in the incidence of any pocket hematoma, CSH and thromboembolic events between the continued and interrupted DOAC strategies [116]. Pooled analyses of the BRUISE CONTROL-2 trial found similar CSH rates with continued versus interrupted DOAC [16]. Until randomized evidence is available, the balanced approach is to omit the morning dose on procedure day and resume 24 h later if hemostasis is secure, avoiding bridging.

## 4. Prevention and Treatment of Pocket Hematoma

### 4.1. Pre-Procedural Measures: Rigorous Assessment on Antithrombotic Strategy

Optimal management of antithrombotic therapy is essential to minimize bleeding risks. According to 2021 EHRA expert consensus [80], the choice of antiplatelet therapy should be individualized based on bleeding and thrombotic risk assessments (Table 1). DAPT is a strong predictor of pocket hematoma with 4–5-fold increased risk [37]; therefore, elective CIED implantation should be delayed for at least one year in ACS patients following bare-metal stent (BMS) placement within 4 weeks or drug-eluting stent (DES) within 6 months (or new-generation DES within 3 months) to allow temporary DAPT discontinuation [75,117]. If delay is not possible, it is proposed that P2Y12 inhibitor should be stopped in eligible patients 5–7 days preoperatively and resume promptly after the procedure while aspirin is maintained [118]. Only when CIED was urgently needed in patients within 6 months of PCI of ACS, or with other high-risk features and in those within 1 month of non-ACS coronary artery disease without high risk was DAPT recommended to continue with the acknowledgement of the risk of increased bleeding. Since the periprocedural cessation of aspirin in patients with coronary artery disease was associated with a 3-fold increase in major adverse cardiac events [119], aspirin monotherapy for secondary prevention does not require discontinuation during most CIED implantations [118,120], whereas aspirin for primary prevention should be stopped 5 days before surgery [118]. Notably, antiplatelet agents independently elevate the risk of pocket hematoma, and this risk is amplified when combined with anticoagulant therapy [34,121,122]. Caution should be exercised when antiplatelets are used with anticoagulants, prioritizing anticoagulant continuation while adjusting antiplatelet levels.

Based on the BRUISE CONTROL trial [4] and meta-analyses [102], uninterrupted OAC is preferred over heparin bridging in patients with moderate-to-high thromboembolic risk (CHA2DS2-VASc ≥ 2) [78,80,118,123,124,125]. The INR was targeted to be ≤3.0, with an ideal operative range of 2.0–2.5. A slightly higher limit of ≤3.5 is acceptable for those with mechanical heart valves. As for DOAC, studies demonstrated that both continued and interrupted DOAC strategies yield comparably low and statistically insignificant rates of clinically significant pocket hematoma [16]. Consequently, the decision to interrupt DOAC therapy should not be applied universally but must be carefully weighed against the patient’s thromboembolic risk profile. For patients with high stroke risk or a high CHA_2_DS_2_-VASc score, a strategy of uninterrupted DOAC therapy may be preferable [16]. Given the relatively rapid onset of action of DOACs, a brief interruption of DOAC therapy without heparin bridging is acceptable for patients at lower risk [38,118], often involving taking the last dose on the morning of the day before surgery and restarting anticoagulation one day post-procedure [126]. In cases of emergent bleeding related to DOACs, specific reversal agents, namely idarucizumab for dabigatran and andexanet alfa for factor Xa inhibitors, have altered the risk–benefit calculus [127].

#### Scores to Assess Pocket Hematoma Risk

The various bleeding scores HASBLED, ORBIT, ATRIA, HEMORR2HAGES and CRUSADE have emerged as reliable predictors of patient’s bleeding risk, but their value to stratify pocket hematoma risk in the setting of uninterrupted OAC or AP during CIED procedure remains controversial.

The HAS-BLED score was reported to predict bleedings during bridging of chronic OAC [128,129,130], but failed to predict the incidence of significant pocket hematoma among patients treated with OAC or aspirin [66]. As for patients with uninterrupted OAC, a pilot study revealed that HEMORR2HAGES was significantly increased in those developing pocket hematoma, regardless of age, gender and procedural INR, whereas no significant differences were found for HASBLED and ATRIA scores [84]. Overall, these two scores were quite appliable and shared some common risk factors such as age, hypertension, renal function, anemia and stroke, but both failed to contain other important factors such as BMI and implantation-related factors. More targeted scores were expected if the relevant risk factors of pocket hematoma had been comprehensively taken into account.

### 4.2. Periprocedural Measures

Strategies to prevent hematoma are focused on not only implanting procedure itself (such as meticulous cautery of bleeding sites, meticulous suture for sub-cuticular layer, use of hemostatic) but also the perioperative management (Table S3).

#### 4.2.1. Surgical Technique

Good surgical technique is the cornerstone of preventing pocket-related complications, including minimized tissue trauma and careful hemostasis [1]. Several specific technical measures contribute to this goal. The use of meticulous electrocautery for hemostasis is widely recommended to control bleeding from small vessels [27]. The wide-awake-local-anesthesia-no-tourniquet (WALANT) technique in CIED implantation with uninterrupted AP therapy has been reported to reduce hematoma size [131]. For venous access, the cephalic vein cutdown technique is prioritized, as it provides controlled access and avoids the risks associated with “blind” subclavian vein puncture. When percutaneous puncture is necessary, ultrasound-guided axillary vein access is superior to the conventional landmark-based approach [73]. Capsulectomy was advised to avoid in order to reduce hematoma formation and infection risk [1,32,132]. Furthermore, if a postoperative hematoma occurs, diagnostic or therapeutic aspiration is contraindicated, given the high risk of inoculating the sterile pocket with skin flora and causing an infection. Evacuation should only be considered in an operating room setting for severe cases with unmanageable pain or threatened wound closure [1]. Regarding wound closure, the use of a non-invasive tissue adhesive device (e.g., ZIP^®^ Surgical Skin Closure) has been shown to significantly reduce pocket closure time and total procedure time compared to standard sutures, without increasing the risk of device pocket infections [133]. In addition, the antibacterial envelope has been reported to be associated with an 82% reduction in the risk of major CIED infection compared to standard care alone among patients who developed a post-procedural hematoma [29], and is thus advocated by the 2021 EHRA consensus for routine use in high-risk patients [80].

#### 4.2.2. Hemostatic Medications

A variety of biodegradable hemostatic materials are available as adjuncts to achieve meticulous pocket hemostasis through distinct mechanisms, including providing a physical matrix for clot formation (e.g., gelatin sponges like Stypro^®^ [17] and oxidized cellulose like Surgicel^®^ [134]), concentrating clotting factors (microporous polysaccharide hemispheres such as MPH powder [135] and PerClot^®^ [136]), or mimicking the final step of the coagulation cascade (fibrin sealants [137]). Several studies, though often retrospective or small-scale, have associated their use with a reduced incidence of CSH by promoting local clotting [135,137,138]. A recent analysis demonstrated a dual benefit of hemostasis and infection prevention of a gentamicin-coated collagen implant (GCCI) at 12 months compared to standard care [139].

However, the data remain heterogeneous, and concerns about foreign-body reactions and infection [136]. Agents that rely on transient vasoconstriction, such as epinephrine or collagen-thrombin mixes, may mask bleeding points and have been linked to higher hematoma rates [140,141]. From the view of EHRA expert consensus statement, no data supporting the routine use of topical hemostatic agents, although they may be useful in selected patients [1,80]. Therefore, the routine adoption of these agents requires further validation from large, randomized controlled trials.

#### 4.2.3. Drainage

The vacuum drain has been applied in surgery, and was once reported to reduce pocket hematoma incidence in CIED implantation as well [142], but there is still debate on its exact effectiveness and secondary risk [80]. Other studies also revealed that the vacuum drainage system brought no significant benefit for reducing hematoma post-CIED procedure [9,17]. Tscholl Verena et al. reported that the use of Polysaccharide Hemostatic System resulted in no significant difference in pocket hematoma, but an augmented incidence of fever and inflammatory markers, and thus terminated the study [136]. A small-caliber “sub-pocket” drain inserted for 12–24 h has been advocated for selected high-bleeding-risk cases, yet its benefit remains unproven [143]. Drainage should, therefore, be reserved for situations with documented intra-operative oozing rather than used prophylactically.

### 4.3. Post-Procedural Measures: Compression Device

Traditional sandbags or adhesive tape dressings often deliver inconsistent pressure and may cause skin tears or allergic reactions. Novel compression devices, including adjustable pneumatic systems with transparent windows for continuous wound assessment [144], specialized compression vests with targeted pressure delivery [145,146], hypothermic compression bandages [147], and mechanical devices with calibrated pressure control [148,149], and several dedicated compression garments (elastic belts, foam-filled pads, vacuum-formed splints) have demonstrated reductions in grade 2–3 hematomas without increasing skin complications [17,150,151,152]. While cost-effectiveness and comparative data remain limited, these devices are a reasonable option for patients at high bleeding risk.

## 5. Main Debate and Limitation

As we have pointed out, the standard definition of pocket hematoma varies a lot in the literature, which may account for the observed difference in the incidence of pocket hematoma in studies with population and procedure heterogeneities. The primary concern about pocket hematoma is to establish a universal definition and comprehensive method of assessment, especially for subcutaneous or intermuscular pocket hematoma. The advancement of technologies has provided new horizons to measure the hematoma, such as the dual-energy CT imaging that was more likely to eliminate metal artifacts in pacemaker leads and potentially showed the margin of pocket hematoma in front of the pulse generator. Besides the size and clinical consequence, there are other characteristics that can be utilized as evaluation parameters, such as the postoperative decrease in hemoglobin level.

In general, a series of risk factors have been recognized significantly relevant with higher incidence of pocket hematoma, including advanced age, low BMI, poor kidney function, complex procedure (replacement/upgrade of CIED) and antithrombotic therapy, especially dual antiplatelet therapy and heparin/LMWH “bridging”. The immediate priority for the next step is to identify the risk factors of pocket hematoma with more effective evidence, and to establish a scoring system to predict the risk of pocket hematoma.

Despite accumulated data, there are still gaps in many topics, including exact timing for surgical intervention, optimal strategies for anticoagulation and antiplatelet therapy during CIED procedure. More large-scale prospective clinical studies with well-defined protocols and interventions are anticipated.

Ultimately, the prevention of pocket hematoma extends beyond avoiding a local surgical complication but is also a critical component in optimizing recovery and enabling efficient healthcare delivery. The growing body of evidence, including recent work demonstrating the feasibility of same-day discharge even after complex procedures like lead extraction in selected patients [153], places a premium on strategies that ensure a complication-free postoperative course. This review synthesizes the evidence on risk factors and preventive measures for pocket hematoma, with the aim of supporting clinicians in achieving excellent outcomes that facilitate such advanced care models.

## 6. Conclusions

With the burgeoning development of CIEDs, the problem of pocket hematoma has been of particular interest since it increases the risk of infection, death and prolongs the hospital stay. Despite controversies, advanced age, low BMI, chronic renal dysfunction, CIED of large size, and anticoagulant or antiplatelet drugs (especially combination) were frequently mentioned as significant risk factors of pocket hematoma in multiple studies. Traditional methods, such as bandage compression and sandbag pressing, are still widely used to prevent pocket hematoma. Alternatively, more effective measures or new compression devices to reduce pocket hematoma incidence are anticipated.

## Figures and Tables

**Table 1:** Perioperative management of anticoagulants and antiplatelet drugs.

Antiplatelet Strategies			
	<1 Month	1–6 Months	>6 Months
Primary prevention of ASCVD	interrupt for 5–7 days		
Secondary prevention of ASCVD	continue ASA		
Non-ACS + PCI without high risk ^a^	continue DAPT	continue ASA, interrupt P2Y12 inhibitors for 5–7 days	
ACS + PCI or with high risk ^a^	continue DAPT	prefer continuing DAPT; may consider interrupting P2Y12 inhibitors if with high risk of bleeding	continue ASA, interrupt P2Y12 inhibitors for 5–7 days
**Anticoagulation Strategy**			
	**Low thrombotic risk (CHA2DS2VASc < 2)**	**Moderate–high thrombotic risk (CHA2DS2VASc ≥ 2)**	
VKA	interrupt without heparin bridging	continue with an INR at the lower end of the target range	
DOAC	consider interrupting based on CrCl and types of DOACs	as operator preference and/or thromboembolic risk	
OAC + AP	continue OAC only	always try to avoid the association based on risk/benefit assessment	

^a^ Prior stent thrombosis on adequate antiplatelet therapy; stenting of the last remaining patent artery; diffuse multivessel disease especially in diabetic patients; creatinine clearance <60 mL/min; ≥3 stents implanted; ≥3 lesions treated; bifurcation with two stents implanted; total stent length >60 mm; treatment of chronic total occlusion.

## Data Availability

No new data were created or analyzed in this study.

## Supplementary Materials

The following supporting information can be downloaded at https://www.mdpi.com/article/10.3390/jcdd12120490/s1, Table S1: Summary of key studies on antithrombotic management and pocket hematoma risk during CIED procedure; Table S2: Bleeding complication rate by AP and/or anticoagulation strategy during the perioperative period; Table S3. Major studies on the prevention measures against pocket hematoma; Table S4. PRISMA 2020 checklist; Table S5. Abbreviation.
