# Supplementary material for: Risk Factors and Preventing Strategies of Pocket Hematoma After Cardiac Implantable Electronic Device Implantation: A Systematic Review
Source: J Cardiovasc Dev Dis. 2025 Dec 12;12(12):490. doi: 10.3390/jcdd12120490 (PMC12734374; doi:10.3390/jcdd12120490)
Supplement: Supplementary file 1 [file jcdd-12-00490-s001.zip › jcdd-3892029-Supplementary-Supply.pdf]

Table S1. Summary of Key Studies on Antithrombotic Management and Pocket Hematoma Risk during CIED Procedure

Author	Year	Case No.	Type of Study	Type of Device	AC/AP Therapy	Definition of Pocket Hematoma	Time of Assessment	Incidence of Pocket Hematoma	Risk Factors (Indicated by Item Numbers) or Conclusions (No Item Numbers)
Goldstein et al[86]	1998	150	Retrospective observational single-center	PPM implantation, revision, or generator replacement	Warfarin (n=37); Control (n= 113): non-anticoagulated, including 8 who stopped warfarin	Bloody Wound Drainage	7-10 days postoperatively	Warfarin=1/37 (2.7%)<br>Control=2/113 (1.8%)	Continuing warfarin (mean INR 2.5) did not increase complications. No hematomas required evacuation. Low bleeding risk with careful technique.
Michaud et al[23]	2000	192	Prospective single-center randomized	PPM or ICD	Group 1: heparin at 6h (n=26);<br>Group 2: heparin at 24h (n=23);<br>Control 1: warfarin alone (n=28);<br>Control 2: no anticoagulation (n=115).	A palpable mass that protruded ≥2 cm anterior to the pulse generator and lead(s), evacuated if tense swelling caused poor capillary perfusion of the overlying skin or severe pain or if the hematoma enlarged progressively.	Not mentioned	Heparin at 6h=23%<br>Heparin at 24h=17%<br>Warfarin alone=4%<br>No anticoagulation=2%	Heparin use led to 20% hematoma risk vs. 4% with warfarin and 2% with no anticoagulation ($p<0.001$). No difference between 6h and 24h heparin initiation.
Al-khadra et al[22]	2003	47	Prospective single-center	New PPM, ICD, or lead revision or generator replacement	Warfarin (n=47)	Not mentioned	At 6 weeks postoperatively	2.1%	No major bleeding or hematomas requiring evacuation. Continuing warfarin is safe with meticulous hemostasis.
Giudici et al[87]	2004	1025	Retrospective	PPM and ICD	Warfarin (INR≥1.5) (n=470);<br>No AC (INR<1.5) (n=555)	Not mentioned	At least 2 weeks after implantation	Warfarin=1.9%<br>INR≥1.5=2.8%<br>INR<1.5=2.2%<br>No AC=1.6%	Continuing warfarin does not increase bleeding risks.
Wiegand, et al[9]	2004	3164	Prospective observational single-center	PPM or ICD	High-dose heparin (n=551);<br>Low-dose heparin (n=765);<br>Phenprocoumon, ASA alone, combined	Pocket bleeding defined as combined end point of intraoperative bleeding or pocket hematoma (any palpable swelling of the	3 months after implantation	Any pocket hematoma=4.9%<br>Severe pocket hematoma=2.21%<br>Reoperation=0.98%	• High-dose heparin (HR=4.2)<br>• Combined ASA and thienopyridine (HR=5.2)<br>• Low level of implanter experience (HR=1.6)

					ASA/thienopyridine, etc.	pacemaker pocket exceeding the size of the generator)			
Marquie et al[68]	2006	228	Retrospective case-control	PPM	With mechanical valves (n=38); matched controls (n=38).<br>With atrial fibrillation (n=76); matched controls (n=76).<br>Heparin (n=89);<br>Control (n=38)	Hemorrhagic AEs were defined as events related to external or internal bleeding or hematomas including bloody effusions in pericardium or pleural space.	30 days post-surgery	Hemorrhagic and procedure-related AEs:<br>Heparin=15.7%<br>Control=1.1%	Post-operative heparin (RR=14). MV patients had higher risk. Hematoma risk was associated with early heparin restart.
Robinson et al[21]	2009	148	Retrospective single-center	PPM or ICD	Pre/postoperative heparin (n=67);<br>No pre/ postoperative heparin (n=7);<br>Pre/no postoperative heparin (n=39);<br>No pre/no postoperative heparin (n=35)	A significant hematoma was defined as a palpable swelling of the pacemaker pocket, exceeding the size of the generator, that required reoperation or interruption of oral anticoagulation.	Between 1~4 weeks following the procedure	Pre/post=22%<br>No pre/ post=29%<br>Pre/no post=8%<br>No pre/no post=9%	• Postoperative LMWH (P=0.001)<br>• INR on the day of surgery (P=0.03)<br>• Male (P=0.05)
Tischenko et al[54]	2009	272	Prospective non-randomized single-center	CIED	Warfarin continued (n=117);<br>Not warfarin (matched control) (n=117);<br>Bridging with dalteparin (n=38)	Considered significant if the hematoma required reoperation and/or transfusion and/or unplanned or prolonged hospitalization and/or interruption of warfarin therapy or incremental outpatient follow-up.	1 week after the procedure	Warfarin=7.7%<br>Control=4.3%<br>Heparin bridging=23.7%	Continuing warfarin is safe with similar hematoma rates to controls (7.7% vs. 4.3%, P=0.41) and lower than bridging (23.7%, P=0.012).<br>• The number of leads (OR 3.3 for each additional lead, P=0.012)
Tolosana et al[2]	2009	101	Prospective randomized single-center	Implant/replacement of a PPM/ICD	Uninterrupted OAC (INR=2±0.3, n=50);<br>Interruption with LMWH bridging (n=51)	Palpable mass that protruded >2 cm anterior to the pulse generator. Evacuated if tense swelling with poor capillary perfusion, progressive enlargement, or severe pain.	At discharge, 15~45 days after the procedure	Pocket hematoma=8% in both groups<br>Reoperation=2%	Maintaining OAC is as safe as bridging with similar hematoma rates (8.0% vs. 7.8%, P=1.00), and reduces hospital stay (median 2 vs. 5 days, p<0.001).
Ahmed et al[89]	2010	459	Retrospective observational	PPM or ICD, generator	Continued warfarin (n=222);	A significant pocket hematoma was defined as a	Within 1 week of	Overall=2.2%	Continuing warfarin with therapeutic INR reduces

			single-center	replacement, or lead revision	Warfarin held with heparin bridging (n=123);<br>Warfarin held without bridging (n=114)	palpable tense swelling causing severe pain that required prolonged hospitalization and/or discontinuation of anticoagulation and/or surgical evacuation and/or blood transfusion.	device implantation	Continued warfarin=0.45%<br>Bridging=5.7%<br>Warfarin withheld without bridging =1.75%	pocket hematoma compared to bridging (0.45% vs. 5.7%); Interrupting warfarin increases thromboembolic events.<br>• AP (ASA or clopidogrel)<br>• heparin<br>• chronic kidney disease (OR=3.84, =0.02)
Chow et al[24]	2010	518	Retrospective single-center	PPM	Patients with peri-operative therapeutic anticoagulation vs. those without.	Based on documented clinical and/or ultrasound diagnosis of wound hematoma by the implanting surgical team, and were sub-classified according to whether they required reoperation for surgical evacuation.	Within 6 weeks after implantation	Total=4.9% (mostly in bridged group)<br>Surgical evacuation=3.8%	• Peri-operative anticoagulation (OR=46.51 , P<0.001)<br>• Acute procedure (OR=4.69, P=0.02)<br>• History of ischemic heart disease
Dreger et al[69]	2010	427	Prospective and retrospective single-center	PPM or ICD	DA-therapy (n=109);<br>Control (ASA alone or no AP) (n=318)	a palpable swelling of the pacemaker pocket exceeding the size of the generator	within 30 days after implantation	Hematoma=0.9% (In all patients, pockets were drained using vacuum drainage systems)	When drainage systems are used, device implantation is safe under continued DA-therapy without significantly increased hematoma risk.
Ghanbari et al[88]	2010	123	Retrospective	CRT-D	High-risk patients: continued warfarin (INR≥2) (n=20); bridging (INR≤2) (n=29).<br>Low-risk patients: cessation of warfarin (n=74).	Palpable mass that protruded >2cm anterior to the pulse generator.<br>Considered significant if the hematoma required evacuation, reoperation, drop in hemoglobin >2 g/dL, blood product transfusion, or interruption of warfarin therapy	at discharge and 15 and 30 days postoperatively	Continued warfarin=5%;<br>Bridging=20.7%;<br>Cessation of warfarin=4.1%	Implantation without warfarin interruption in patients at high risk of thromboembolic events is a safe alternative to bridging therapy (5.0% vs. 20.7%, p=0.03).
Kutinsky et al[26]	2010	935	Prospective observational	Implantation of a PM or an ICD	Groups based on medication used:	palpable swelling with fluctuance over the device	Not mentioned	9.5%	• Device indicated for primary prevention of

			single-center		clopidogrel, heparin, etc.; no formal control group	generator. Type 1 did not extend beyond 1 cm past the device margin, and type 2 extended beyond 1 cm past the device margin and/or caused significant strain on incision site.			sudden cardiac death (OR=1.62; P=0.037)<br>• Clopidogrel use (OR=2.32; P=0.0008)<br>• Intravenous heparin (OR=3.62; P<0.0001)
Przybylski et al[60]	2010	247	Prospective two-center	CIED	ASA alone (n=194)<br>DAPT (n=53)	Major:<br>bleeding requiring blood transfusion, surgical intervention, pericardiocentesis, pocket revision or prolonging hospitalization.<br>Minor:<br>small pocket hematomas not requiring treatment or prolonging hospitalization; subcutaneous hematomas and local ecchymosis	12 months after implantation	Overall bleeding complications =16.2%<br>Major=3.6%<br>Minor=14.2%<br>ASA alone=13.9%<br>DAPT=24.5%	DAPT does not increase major bleeding risk but raises minor complications; DAPT can be continued with moderate risk.
Pakarinen et al[25]	2010	567	Retrospective single-center	Cardiac rhythm management device	Warfarin and LMWH were discontinued before device implantation, and resumed respectively after 24h, 12-24h post procedure. AP therapy was continued.	Pocket hematoma or bleeding was defined as swelling of the pocket with the need for reoperation or for prolonged hospital observation.	3-month follow-up	Overall=3.2%<br>Hematomas requiring surgical evacuation=1.2%	All patients suffering pocket hematoma or bleeding were on AP or AC before implantation.<br>• Operated by trainees (P=0.037)
Poole et al[61]	2010	1031	Prospective multi-center (REPLACE Registry)	PPM or ICD or replacements and upgrade procedures	No special intervention on AC and AP therapy	Hematoma adjudicated by the Clinical Events Committee	6 months after replacement	Hematomas requiring evacuation 0.7%-1.5%;<br>Mild hematomas 3.5%-4.3%	/
Thal et al[75]	2010	200	Retrospective observational single-center	PPM or ICD	Warfarin only (n=39)<br>ASA+warfarin (n=13)	Not mentioned	6 weeks after implantation	Overall=3.5%	DAPT significantly increases hematoma risk.

					Clopidogrel+warfarin (n=1)<br>DAPT+warfarin (n=5)<br>ASA only (n=82)<br>Clopidogrel only (n=2)<br>DAPT (n=15)<br>None (n=43)				Warfarin alone does not significantly increase risk.
Tompkins et al[65]	2010	1388	Retrospective chart review	PPM or ICD	No AP/ AC (n=255);<br>ASA alone (n=536);<br>DAPT (n=139);<br>Warfarin held (INR <1.5) (n=258);<br>Warfarin continued (INR≥1.5) (n=46);<br>Heparin bridge (n=154)	A significant bleeding complication was defined as need for pocket exploration or blood transfusion; hematoma requiring pressure dressing or change in anticoagulation therapy; or prolonged hospitalization.	Within 6 weeks of implantatio n	bleeding complications =5.1%	• DAPT (OR 3.84; P=0.040)<br>• Heparin (OR 9.88; P<0.0001)<br>• Warfarin INR≥1.5 (OR 5.64; P=0.034)<br>• Male (OR 2.51; P=0.025)<br>• African American (OR 0.51; P=0.025)<br>• Weight (OR 0.97; P=0.001)<br>• Procedure time, per 10 min (OR 1.04; P=0.006)
Cano et al[27]	2011	849	Prospective observational single-center	CIED	SAPT (n=220);<br>DAPT (n=60);<br>OAC+bridging+AP therapy (n=40);<br>no AP or OAC (n=375);<br>OAC+ bridging (n=154)	Pocket hematoma was defined as a palpable mass that protruded 2 cm anterior to pulse generator	within7 days of implant	Total=6.2%<br>SAPT=3.2%<br>DAPT=13.3%<br>OAC+ bridging+AP therapy=15%<br>no AP or OAC =2.4%<br>OAC+bridging=14.9%	• Obesity (OR 0.38; P =0.007)<br>• Renal insufficiency (OR 2.44; P=0.027)<br>• DAPT (OR 8.27; P<0.001)<br>• Enoxaparin bridging (OR 7.10; P<0.001)
Cheng et al[6]	2011	100	RCT	PPM or ICD	Continued warfarin (n=50);<br>Warfarin held with heparin bridging (n=7);<br>Warfarin held without bridging (n=43)	Significant bleeding was defined as extracardiac bleeding or pocket hematomas that required additional intervention and/or temporary discontinuation of anticoagulation therapy.	7-10 days and 4-6 weeks after the procedure	Continued warfarin=0%<br>Heparin bridging=28.6%<br>Discontinued warfarin=0%	Trend towards reduced complications with continued warfarin; not statistically significant.

Li et al[85]	2011	766	Retrospective single-center	PM, ICD or CRT	Interrupted warfarin (n=243);<br>Continued warfarin (n=324);<br>LMWH bridging (n=199)	A palpable hematoma, and/or refractory pain, or threatened integrity of the incision was defined as having a moderate or severe hematoma; Ecchymosis was not defined as hematoma.	Within 30 days after the procedure	Total=3.8%;<br>Interrupted warfarin=2.1%;<br>Continued warfarin=3.7%;<br>LMWH bridging=7.0%	Continuation of OAC with INR <2.5 does not increase bleeding risk; heparin bridging increases bleeding.
Tompkins et al[5]	2011	1440	Retrospective	PPM and ICD	No special intervention on AC and AP therapy	Hematoma as a component of composite end point of bleeding complications (including pocket exploration, blood transfusion, medication discontinued, prolonged hospitalization)	within 60 days after procedure	bleeding complications = 5.7%	• Clopidogrel (OR 1.85; P=0.043)<br>• Male (OR 0.35; P =0.002)<br>• Increased body weight (OR 0.98; P=0.030)<br>• CKD stage 4 (OR 3.1; P=0.01)<br>• CKD stage 5 (OR 7.3; P<0.0001)<br>• Heparin (OR 3.7; P <0.0001)
Boulé et al[67]	2012	202	Retrospective case-control study	PPM/ICD implantation or generator replacement	Clopidogrel (n=101);<br>Control (included patients treated with ASA alone as well as patients taking no AP) (n=101)	A pocket hematoma was defined as a palpable mass that protruded >2 cm anterior to the pulse generator.<br>A significant bleeding complication was defined as pocket hematoma requiring surgical evacuation or prolonged hospitalization, hemothorax, pericardial effusion, or tamponade.	Not mentioned	Bleeding complication=7.9%<br>Pocket hematoma=6.4%	• Clopidogrel (OR 3.7, P=0.03)<br>• Subclavian venous puncture (OR 3.44, P=0.03)
Healey et al[107]	2012	4591	Randomized trial (RE-LY sub-analysis)	Procedure including PPM/ICD and non-cardiac surgery	Dabigatran 110mg (n=1487);<br>Dabigatran 150mg (n=1546);<br>Warfarin (n=1558)	Major bleeding was defined as a reduction in hemoglobin of at least 20 g/L, transfusion of at least 2 U of blood, or symptomatic bleeding into a critical area or organ.	From 7 days before the procedure until 30 days after	Major bleed: 3.8% vs. 5.1% vs. 4.6%;<br>Minor bleed:8.1% vs. 9.0% vs. 7.8%	Dabigatran similar to warfarin in periprocedural bleeding and thromboembolic events; allows shorter interruption time.

Lee et al[93]	2012	260	Retrospective single-center	PPM	DAPT (n=25); ASA (n=54); Clopidogrel (n=4); Interruption of wafarin with heparin bridging (n= 14)	A significant bleeding complication requiring pocket exploration due to increasing size despite of compression dressing or a blood transfusion of more than 2 pints because of a decreased hemoglobin >2 g/dL after a procedure or a change in vital signs.	within 6 weeks of the procedure	Significant bleeding complication=3.1%	Hematoma after PPM implantation was rare. Heparin bridging significantly increased bleeding complications (p<0.0001). AP did not significantly increase risk.
Airaksinen et al[36]	2013	213	Randomized multi-center (FinPAC study)	PPM or ICD	Uninterrupted warfarin (n=106)<br>Interrupted warfarin (n=107)	Any palpable mass or ecchymosis in the pocket area. A hematoma > $100cm^2$ was considered as a large hematoma.	At discharge and 30-90 days after the procedure	Any pocket hematoma: Uninterrupted warfarin =33%; interrupted warfarin =40%;<br>Large hematoma: 6% in both groups	Uninterrupted warfarin is safe with no increased bleeding risk during device implantation.
Birnie et al[4]	2013	668	Multi-center single-blind RCT (BRUISE CONTROL)	PPM or ICD	Continued warfarin (n= 343);<br>Heparin bridging (n=338)	CSH: hematoma requiring further surgery, resulting in prolongation of hospitalization, or requiring interruption of OAC.	During the hospitalization and at 1 to 2 weeks of follow-up	Continued warfarin=3.5%<br>Heparin bridging=16%	• Continued warfarin (vs. heparin bridging) (RR=0.16, P<0.001)<br>• Diabetes Mellitus (RR=0.48, P = 0.01)<br>• ASA (RR=2.04, P = 0.01)
Chen et al[52]	2013	1093	Retrospective	CIED	No antithrombotic (n=512);<br>Continuing AP (n=477);<br>Warfarin with/without bridging (n=104)	A swelling and painful mass with ecchymosis formation and extending the margin of generators	during hospitalization and at least 6-8 weeks after discharge	Overall=3.7%	• Moderate to severe thrombocytopenia (OR=4.02; P=0.007)<br>• DAPT (OR=5.24; P=0.004)<br>• Heparin bridging (OR=6.04; P<0.001)
Jennings et al[105]	2013	257	Comparative study	CIED	Uninterrupted dabigatran (n=48);<br>Interrupted dabigatran (morning dose omitted) (n=14);<br>Uninterrupted warfarin (n=195)	Bleeding complications including any clinically evident pocket hematoma (visible swelling, fluctuance, drainage, excessive tenderness) or pericardial	From the post-procedure period through the 4- to 6-week	Uninterrupted dabigatran=1/48 (2.1%);<br>Interrupted dabigatran=0%;	Bleeding risk is similar between uninterrupted dabigatran and warfarin (P=0.69); combination with antiplatelet drugs increases risk.

						effusion/tamponade documented by echo	outpatient visit	Uninterrupted warfarin=9/195 (4.6%)	
Özcan et al[10]	2013	574	Retrospective-prospective single-center	PPM	ASA only (n=196);<br>Clopidogrel only (n=37);<br>DAPT (n=24);<br>Discontinued warfarin (n=59);<br>LMWH bridging (n=48);<br>No AP/AC (n≈209)	Hematoma was defined as a swelling of pacemaker pocket with or without tenderness and pain.	4-6 weeks after the procedure	Overall=3.6%<br>Surgical evacuation=1.6%<br>ASA=1.5%<br>Clopidogrel=5.4%<br>DAPT=4.2%<br>Warfarin=20.3%<br>Heparin bridging=6.1%	• Warfarin (median INR=1.3) (P<0.001)
Palmisano et al[15]	2013	2671 procedures in 2,648 patients	Retrospective observational dual-center	De novo device implantation, elective generator replacement, or pacing system upgrade	No special intervention of AP/AC	Pocket hematoma was defined as a palpable mass that protruded >2 cm anterior to the pulse generator.	Median follow-up of 27 months	0.37%	• OAC (OR=8.98; P<0.001);<br>• DAPT (OR=7.30; P<0.001);<br>• LMWH bridging in OAC patients (OR=3.38, P=0.037)
Beyer-westendorf et al[113]	2014	863 procedures in 595 patients	Prospective observational	Surgical/interventional procedures	DOACs	Major bleeding (ISTH criteria): overt bleeding plus ≥1 of: Hb drop ≥20 g/L, transfusion ≥2 units, surgical revision, bleeding into a critical site, or fatal bleeding.	Until Day 30 ± 5 after the procedure	DOAC continued=0.5%;<br>DOAC interrupted without bridging= 0.5%;<br>Any heparin bridging vs no bridging: 2.7% vs 0.5% (P = 0.010)	Short interruption of DOAC without heparin bridging is safe.<br>• Major procedure (OR=16.8)<br>• Heparin bridging (OR=5.0)
Chen et al[79]	2014	342	Prospective observational	CIED	DAPT (n=86);<br>DAPT discontinued and replaced with LMWH (n=85);<br>Control (n=171)	A pocket hematoma was defined as a palpable mass protruding >2cm past the anterior margin of the pulse generator.	6 months after discharge	DAPT=3.49%<br>LMWH=16.47%<br>Control=1.17%	• LMWH (RR=0.054, P<0.001)
De Sensi et al[84]	2014	35	Pilot study	CIED	Uninterrupted OAC (n=35)	Hematomas were described as suffusions or ecchymosis in the pocket area, requiring	Within 1 week after	14.3%	HEMORR2HAGES bleeding score is a significant predictor of

						frequent ambulatory follow-up and temporary reduction of OAC usual dosage.	the procedure		pocket hematoma while on OAC.
Kosiuk et al[111]	2014	236	Case control study	Cardiac rhythm device (CRD)	Uninterrupted warfarin (n=118);<br>Interrupted dabigatran (n=118)	Pocket hematoma causing prolongation of hospitalization, discontinuation of the AC or the necessity for transfusion of blood products.	Within 30 days after procedure	Warfarin=8%<br>Dabigatran=3%	Dabigatran may have lower bleeding complications and shorter hospitalization compared to uninterrupted warfarin.
Kosiuk et al[104]	2014	176	Prospective observational	Cardiac rhythm device (CRD)	Dabigatran (n=93);<br>Rivaroxaban (n=83)	Pocket hematoma causing prolongation of hospitalization, discontinuation of the AC or the necessity for transfusion of blood products.	within 30 days after procedure	Dabigatran=2.2%<br>Rivaroxaban =3.6%	Bleeding complications are rare with DOACs; rivaroxaban may have a trend toward higher bleeding risk compared to dabigatran.
Guo et al[49]	2014	972	Retrospective	CIED	No special intervention of AP/AC	Severe pocket hematoma was palpable swelling of the pocket causing pain and prolonged hospitalization, whether or not accompanied by anticoagulation treatment cease, reoperation, evacuation, or blood transfusion	3 months after the procedure	2.2%	• Low BMI (OR=0.32, P=0.020)<br>• DAPT (OR=18.01, P<0.001)<br>• Renal insufficiency (OR=0.98, P=0.030)<br>• Complex devices (OR=8.51, P<0.001)
Nammas et al[66]	2014	447	Post hoc analysis of randomized trial<br>multi-center (based on FinPAC trial)	Cardiac rhythm management device	OAC (n=213);<br>ASA (n=128);<br>No antithrombotic (n=106)	The primary outcome was a significant pocket hematoma (>100 cm^2 in area) and other bleeding complications. Major bleeding was defined as any bleeding or pocket hematoma that required an additional intervention.	At discharge and 1 month after the procedure	OAC=5.6%<br>ASA=5.5%<br>No antithrombotic =0.9%	OAC group:<br>• post-procedural INR (HR 2.6, P=0 .045)<br>ASA group:<br>• the duration of the procedure (HR 1.01, P=0.05)
Schulman et al[97]	2014	171	RCT<br>open-label	PPM or ICD	Interrupted warfarin with LMWH bridging (n=85);<br>Reduced-dose warfarin (n=86)	Pocket hematoma was defined as palpable swelling of the pocket exceeding the size of the generator.	After 3±1 weeks and at 3 months after the procedure	Pocket hematoma:<br>- LMWH=4.7%<br>- warfarin=3.5%<br>Severe pocket hematoma:	The reduced-dose warfarin aiming for an INR≤1.7 on the day of device implantation had similar safety with

						Severe pocket hematoma was defined when there was in addition one or more of pain, prolonged hospitalization or requirement for re-operation.		- LMWH=1.2%<br>- warfarin=1.2%	regards to pocket hematoma as interrupted warfarin with preoperative LMWH bridging.
Cano et al[90]	2015	278	Prospective observational single-center	CIED	Uninterrupted OAC (INR 2-4):<br>- high thromboembolic risk (117);<br>- low thromboembolic risk (161).<br>No intervention on AP therapy	Pocket hematoma was defined as a palpable mass protruding at least 2cm anterior to the pulse generator.<br>Severe pocket hematoma was defined if: required surgical evacuation, or blood transfusion, or prolonged hospital stay, or hospital re-admission.	After 7–10 days after the procedure	Pocket hematoma=2.9%<br>Severe pocket hematoma=1.4%	Continuation of OAC is safe.<br>• CHADS2 score (OR 1.83, P=0.017)
Dai et al[12]	2015	364	Prospective observational single-center	CIED	DAPT (n=31);<br>ASA only (n=124);<br>no AP (n=209)	Pocket hematoma was swelling and a painful mass with ecchymosis formation extending the margin of generators.	In seven days after implantation	Bleeding complications=4.12 %;<br>hematoma=3.8%	• Dual AP therapy (OR 3.70; P = 0.01)
Ghanbari et al[122]	2015	25180	Retrospective analysis of registry data	Primary de-novo ICD	Control: warfarin alone (n=5264);<br>SAPT (n=6538);<br>DAPT (n=3414);<br>Warfarin+SAPT (n=7994);<br>Warfarin+DAPT (n=1970)	Device-related bleeding was based on ICD-9 codes of hematoma complicating a procedure, hemopericardium/tamponade, pericardiocentesis for tamponade, hemothorax; plus any re-operation for bleeding.	Within 30 or 90 days	30-day=0.8%;<br>90-day=1.2%;<br>Warfarin alone=0.6%;<br>SAPT=0.7%;<br>DAPT=0.9%;<br>Warfarin+SAPT=1.0%;<br>Warfarin+DAPT=1.1%	Warfarin alone has lowest bleeding risk.<br>Combinations increase bleeding without reducing thromboembolic events.<br>Major procedure, diabetes, renal failure and higher $CHADS_2$-VASc also associated with excess bleeding.
Melton et al[3]	2015	380	Retrospective single-center cohort study	PPM or ICD	Uninterrupted OAC (n=80):<br>- Warfarin (n=57)<br>- Dabigatran (n=14)	Pocket hematoma was identified by searching the patient's medical record during hospitalization and	Within 30 days of implantation	Total=9.7%<br>OAC=21.3%<br>No AC=7.7%	• Device type, either ICD or PPM (P<0.001)<br>• Rivaroxaban (P=0.006)<br>• ASA (P=0.035)<br>• Therapeutic OAC

					- Rivaroxaban (n=9)<br>No AC (n=300)	clinic follow-up, and was classified as clinically significant if requiring additional intervention such as rehospitalization, antibiotic therapy, surgical intervention, or discontinuation of OAC.		Clinically significant=1.3%	(P=0.001)
Amara et al[81]		2016	20	Retrospective multi-center	PPM or ICD implantation or generator replacement	ASA+ticagrelor (n=20)	A significant bleeding complication was defined as pocket hematoma requiring a surgical evacuation or prolonged hospitalization, hemothorax, pericardial effusion or tamponade.	at 1 month post-procedure	pocket hematoma =5%	Ticagrelor treatment at the time of heart rhythm device procedures does not seem to be associated with an increased risk of significant bleeding complications.
Deharo et al[38]		2016	723	Prospective multi-center (ESS-PREDI)	CIED	VKA alone (n=210)<br>DOAC alone (n=71)<br>ASA alone (n=239)<br>P2Y12 inhibitor alone (n=33)<br>VKA+AP (n=76)<br>DOAC+AP (n=26)<br>ASA+P2Y12 inhibitor (n=68)	Pocket hematoma was defined as a palpable mass protruding ≥2 cm anterior to the CIED pocket; it was considered minor when no treatment was needed other than observation.	Not mentioned	Severe pocket hematoma=1.0%<br>Minor pocket hematoma=8.4%	DOACs associated with lower bleeding complications compared to other antithrombotic therapies.
Madan et al[112]		2016	133	Retrospective single-center cohort study	PPM or ICD	Uninterrupted warfarin (n=86);<br>Interrupted dabigatran (n=47)	Pocket hematoma: a palpable and visible soft mass in the pacemaker pocket; only grades 1 – 3 counted as events.<br>Grade 0: skin ecchymosis or minimal hematoma; Grade 1: visible/palpable but < generator size; Grade 2: greater than the size of the generator; Grade 3: required evacuation.	1-month post-procedure	Warfarin=7% (all grade 1, none evacuated);<br>Dabigatran=0%<br>P=0.09	Interrupted dabigatran appears at least as safe as uninterrupted warfarin for device implantation, with a trend toward fewer pocket hematomas.

Sridhar et al[40]	2016	85276	Retrospective cohort study	ICD and CRT-D	Hematoma group (n=2,233);<br>Control: No hematoma group (n=83,043)<br>No special intervention on AC/AP therapy	ICD-9 codes (99,811e99813: Hemorrhage or hematoma or seroma complicating a procedure)	During the hospital stay	2.6%	• Increased age (P<0.001)<br>• Congestive heart failure (OR=1.86, P<0.001)<br>• Coagulopathy (OR=2.3, P<0.001)<br>• Renal failure (OR=1.52, P<0.001)<br>• Peripheral vascular disease (OR=1.4, P<0.01)
Tolat et al[82]	2016	748	Retrospective single-center	Outpatient ICD	Warfarin (n=230);<br>No AC (n=518)	Significant pocket hematoma was defined as pocket hematoma requiring evacuation, hospitalization, anticoagulation cessation.	At discharge and 30 days after the procedure	Warfarin=3.5%<br>No AC=0.4%	• Wafarin (OR=17.48, P=0.002)
Masiero et al[42]	2017	2500	Prospective observational multi-center (SIMPLE trial)	ICD	No special intervention	The clinically significant pocket hematoma defined as one requiring re-operation (hematoma evacuation) or interruption of OAC or AP therapy	At discharge and at the 30-day post-surgical follow-up visit	2.2%	• Older age (OR=1.03)<br>• History of stroke (OR=2.47)<br>• Upgrade from a PPM to ICD (OR=2.52)<br>• Sub-pectoral ICD (OR=2.00)<br>• Heparin bridging (OR=2.65)
Koh et al[48]	2017	1091	Retrospective single-center cohort	CIED	No special intervention	CSH: a swelling of the device pocket resulting in the need for prolonged admission, which includes cases requiring re-operation.	In-hospital stay only (mean 9.2 days; median 6 days)	Overall hematoma=5.6%;<br>CSH=1.1%	• CKD stage 2 (OR=2.93, P = 0.034) and stage 3 (OR=3.39, P = 0.021);<br>• Heparin/therapeutic enoxaparin (OR=3.15, P = 0.018);<br>• DAPT (OR=2.95, P = 0.026);<br>• BMI 25 – 29.9 (OR=0.52, protective)
Demir et al[53]	2017	232	Retrospective single-center	CIED	Clopidogrel (n=12)<br>ASA (n=73)<br>ASA+clopidogrel (n=29)<br>Warfarin (n=34)	Minor hematomas: cause local pain and could be managed conservatively without requirement of blood transfusion or	Not mentioned	2.6%	• Baseline platelet level (OR= 0.974; P=0.025)<br>• ASA + clopidogrel use (OR= 20.410; P= 0.002)

					Warfarin+ASA (n=21)<br>No AP/AC as control (n=63)	interruption of AP/AC.<br>CSH: necessitating blood transfusion, interruption of AP/AC, prolonged hospitalization and further pocket surgery.			
Essebag et al[108]	2017	611	Post-hoc sub-analysis of a randomized controlled trial (RE-LY trial)	CIED	Dabigatran 110 mg (n = 216);<br>Dabigatran 150 mg (n = 194);<br>Warfarin (n = 201), of whom 37 (18.4%) received heparin bridging	Pocket hematoma: Any bleeding at the device pocket site;<br>Major bleeding: Hemoglobin drop ⩾20 g/L, transfusion ⩾2 units, or symptomatic bleeding into a critical area;<br>Minor bleeding: All other bleeding events.	30 days post-procedure	Dabigatran (both doses)=2.20%;<br>Warfarin overall=3.98%;<br>Warfarin with heparin bridging=10.8%;<br>Warfarin without bridging=2.4%	Short-term interruption of dabigatran is safe, with similar pocket hematoma rates compared to warfarin, and lower than warfarin with heparin bridging.
Malagù et al[96]	2017	1035	Single-center observational	First implantation of CIED	Standard Management (LMWH bridging) (n=522);<br>No-bridge (n=513)	Clinically relevant pocket hematoma was defined as device pocket hematoma determining prolonged hospitalization (>2 days) or requiring surgical reintervention.	Followed at least 30 days	Total=4.1%;<br>LMWH bridging=6.5%;<br>No-bridge=1.6%	• Coronary artery disease (OR= 2.43; P=0.009);<br>• Heparin (OR=3.48; P=0.003);<br>• No-bridge (OR= 0.33; P=0.009)
Bai et al[47]	2017	339	Retrospective single-center	PPM	No special intervention	Pocket effusion or hematoma: any palpable fluid/blood collection in the generator pocket that required prolonged stay or re-operation.	In-hospital stay	Overall=9.73%	• History of allergy<br>• Low BMI<br>• Large-size device<br>• Diabetes mellitus
Ishibashi et al[130]	2017	300	Retrospective observational	CIED	No-AT (n=129);<br>OAC (n=89);<br>SAPT (n=49);<br>OAT+SAPT (n=20);<br>DAPT (n=10);<br>Triple AT (n=3)	Pocket hematoma: bleeding not requiring additional intervention but requiring treatment with compress.<br>CSH: bleeding requiring surgical intervention, prolongation of hospitalization, interruption	Not mentioned	No-AT=3.1%;<br>OAC=6.7%;<br>SAPT=4.1%;<br>OAC+SAPT=25%;<br>DAPT group =0%;<br>Triple AT=0%	• Valvular heart disease (OR=7.2, P = 0.015)<br>• High HAS-BLED score (OR=2.5, P= 0.014)

						of AT, and blood product transfusions.			
Zaca et al[94]	2017	569	Prospective observational multi-center (retrospective pilot phase)	CIED	VKA held, no bridging (n=9)<br>DOAC held, no bridging (n=14)<br>VKA uninterrupted (n=172)<br>DOAC uninterrupted (n=41)<br>SAPT (n=213)<br>DAPT (n=47)<br>Heparin bridging (n=73)	Clinically significant pocket hematoma was defined as one resulting in prolonged hospitalization and/or requiring interruption of antithrombotic therapy and/or requiring further surgery and/or requiring transfusion.	1 month post-procedure	VKA held, no bridging=0%<br>DOAC held, no bridging=0%<br>VKA uninterrupted=0%<br>DOAC uninterrupted=2.4 %<br>SAPT= 0%<br>DAPT=4.2%<br>Heparin bridging=12.3%	Heparin bridging was associated with the highest incidence of clinically significant pocket hematoma (12.3%).
Pecha et al[95]	2018	104	Retrospective single-center Non-randomized Cohort study	Submuscular ICD or CRT-D	Continued phenprocoumon (n=46);<br>Heparin bridging (n=58)	Clinically relevant pocket hematoma: required surgical revision	Day 1 and before discharge; additional follow-up at 1 and 6 months	Heparin bridging=13.8%;<br>Continued phenprocoumon=2. 2%<br>P=0.04	Continued OAC is associated with a lower risk of bleeding complications compared to heparin bridging therapy.
Afzal et al[83]	2018	137	Retrospective single-center	Subcutaneous-ICD	Continued warfarin (n=24);<br>Non-warfarin (n=113)	CSH: detected within 24 hours of the procedure and led to either alteration of OAC or AP management, prolonged length of stay, wound dehiscence, or surgical evacuation.	7-10 days post procedure	Warfarin=25%<br>Non-warfarin=1.5%	• INR>1.8
Birnie et al[16]	2018	662	Prospective multi-center open-label RCT (BRUISE CONTROL-2)	CIED	Continued DOAC (n=328);<br>Interrupted DOAC (n=334)	CSH: a hematoma requiring re-operation, and/or resulting in prolongation of hospitalization, and/or requiring interruption of oral anticoagulation.	In hospital and 1-2 weeks after surgery	Continued DOAC=2.1%;<br>Interrupted DOAC=2.1%	No significant difference in CSH risk between continued and interrupted DOAC strategies; both are safe.
Ricciardi et al[114]	2018	101	Prospective open-label	CIED	Uninterrupted DOAC (n=50);	CSH: a hematoma requiring re-operation, prolonging	In-hospital + outpatient	Interrupted DOAC=0%;	Uninterrupted DOAC therapy appears as safe as

			randomized single-center Pilot trial		Interrupted DOAC (n=51)	hospitalization ⩾24 h, or forcing AC interruption	visits at 2–3 weeks and 2–3 months	Uninterrupted NOAC=2.0% (P=0.320)	interrupted therapy and does not increase bleeding complications.
Sheldon et al[76]	2018	200	Retrospective dual-center	Subcutaneous ICD	Interrupted AC with bridging (n=12);<br>Interrupted warfarin without bridging (n=13);<br>Uninterrupted warfarin (n=13);<br>Interrupted DOAC without bridging (n=13);<br>Uninterrupted DOAC (n=2);<br>Clopidogrel (mainly DAPT, n=14) vs no clopidogrel (n=186)	A hematoma was a device site blood accumulation requiring surgical evacuation, extended hospital stay, or transfusion	52 days after implantation	Overall=5% (6 required evacuation)<br>Interrupted AC with bridging=33%;<br>Interrupted AC no bridging=0%;<br>Uninterrupted warfarin=15%;<br>Any bridging=7%;<br>Clopidogrel vs no clopidogrel: 28.6% vs 3.2 % (P<0.0001)	• Any bridging AC (OR 10.3, P=0.01)<br>• Clopidogrel (mainly DAPT) (OR 10.0, P=0.01)<br>• Uninterrupted warfarin without bridging (OR 11.1, P=0.013)
Essebag et al[121]	2019	681	Secondary analysis of RCT (based on BRUISE CONTROL)	CIED	AP therapy (n=280);<br>No AP therapy (n=401)	CSH: a hematoma requiring further surgery, resulting in prolongation of hospitalization⩾24 h), or requiring interruption of oral anticoagulation therapy.	In-hospital + 30-day clinical follow-up	AP group=12.9%;<br>No AP group=7.5% (P=0.02)	Concomitant AP therapy increases the risk of CSH. Deprescribing AP therapy may reduce hematoma risk.
Ferretto et al[34]	2020	500	Retrospective	CIED	VKA (n=68);<br>LMWH (n=111);<br>DOACs (n=27);<br>AP (n=262);<br>DAPT (n=50);<br>SAPT+AC (n=64);<br>DAPT+AC (n=12)	Pocket hematoma: any large effusion leading to swelling and causing functional impairment or pain requiring any of the following conditions: pocket revision; prolongation of hospitalization; interruption of AC; decrease in hemoglobin value >2.0 g/dl.	Before discharge and at 1-month follow-up visit	Overall=4.6%<br>LMWH=11.0%<br>Replacement=4.4%	• DAPT+AC (OR 6.3);<br>• LVEF<30% (OR 7.4);<br>• LMWH (OR 3.8)

Notaristefano et al[57]	2020	850	Prospective	CIED	SAPT (n=257);<br>DAPT (n=55);<br>DOAC (n=176);<br>VKA (n=68);<br>Heparin (n=54);<br>Triple therapy (n=24);<br>No AP/AC (n=208)	CSH: a palpable and painful swelling in the pocket area and associated with at least one of the following: prolonged hospitalization; interruption of AC therapy; reoperation.	10 days after the procedure	Overall=7.3%<br>SAPT=7.4%<br>DAPT=18.2%<br>DOAC=4.5%<br>VKA=10.3%<br>Heparin=18.5%<br>Triple therapy=20.8%<br>No AP/AC=1.0%	• Procedure type<br>• Antithrombotic treatment
Fensman et al[58]	2022	5918	Prospective	CIED	ASA (n= 2590);<br>Clopidogrel (n= 110);<br>DAPT (n=492);<br>No AP (n=2726);<br>Heparin (n=179);<br>Triple therapy (n=56)	CSH: a hematoma requiring re-intervention, prolonged hospitalization, hospital re-admission, or additional out-patient visits. Hematomas with no treatment consequences were not included (grade 3).	30 days post CIED procedure	Overall=2.5%<br>ASA=2.8%<br>Clopidogrel=1.8%<br>DAPT=6.3%<br>No AP=1.5%<br>Heparin=5.6<br>No heparin= 2.4%	• ASA (aOR=1.8)<br>• DAPT (aOR=3.9)<br>• heparin (aOR=2.1)<br>• INR≥2.0 (aOR=2.0)<br>• Operated by low-volume operators (aOR=2.7)<br>• CRT (aOR=2.0) or dual-chamber defibrillator (aOR=2.1)
de Heide dt al[115]	2022	283	Retrospective single-center	CIED	Continued VKA (n=202);<br>Interrupted DOAC (n=81)	CSH: required re-operation, or prolonged hospitalization >24 h, or forced interruption of OAC	Within 30 days post-procedure	VKA group=2.5%<br>DOAC group=0%<br>(P=0.33)	Risk of pocket hematoma and thromboembolism is low and comparable between interrupted DOAC and continued VKA strategies.
Tarakji et al[77]	2022	6800	Prospective multicenter randomized single-blinded (analysis of WRAP-IT trial)	Secondary CIED procedures or initial CRT-D implantation	AP alone (n=4109);<br>AC alone (n=2968);<br>both (n=1249);<br>neither (n=972)	Included implant site hematoma, incision site hematoma, medical device site hematoma	Within 30 days	Overall=2.2%<br>AC alone=2.5%<br>both=4.56%<br>neither=0.82%	• AC use (HR=2.44)<br>• Lower BMI (HR=1.06)<br>• History of valve surgery (HR=2.11)
Creta et al[110]	2024	861	Multicenter propensity-matched study	CIED	Continued VKA (n=287)<br>Interrupted DOAC (n=287)	Device-pocket hematoma was defined as any palpable swelling anteriorly or laterally to the pulse generator;	Within 30 days post procedure	Overall=7.1%<br>CSH=2.7%<br>Continued VKA=5.2%	• Male (OR=2.3)<br>• Continued DOAC (OR=2.32)<br>• DAPT (OR=3.76)

					Continued DOAC (n=287)	CSH: hematoma requiring re-operation, or prolongation/re-hospitalization ⩾24 h, or interruption of anticoagulation ⩾24 h		Interrupted DOAC=6.3% Continued DOAC=9.8%	
Crider et al[109]	2025	214	Retrospective single-center cohort study	CIED placement or revision	DOAC interrupted (n = 191); DOAC continued (n = 23)	CSH: a hematoma within the medical record and requiring reoperation and/or prolonged interruption of AC.	In-hospital + 30-day post-operation	Overall=8.9%; Interrupted DOAC=9.4%; Continued DOAC=4.3%	Interrupted DOAC strategy may be common, but no significant difference in outcomes compared to continued DOAC.
Pepplinkhuizen et al[63]	2025	347	Retrospective single-center cohort study	de-novo subcutaneous-ICD implantation	VKA continued (n=22); VKA interrupted (n=47); DOAC continued (n=2); DOAC interrupted (n=13); DAPT (ticagrelor-based n=12; clopidogrel-based n=28); SAPT or no AP as reference	CSH: an accumulation of blood at the pocket site, necessitating applying a pressure bandage, surgical evacuation, drain insertion, change in medication or extended hospital stay, or blood transfusion.	30 days post implant	Overall=5.2%; VKA continued=27.3%; VKA interrupted=4.3%; DOAC continued=50%; DOAC interrupted=7.7%; DAPT-ticagrelor=33.3%; DAPT-clopidogrel=3.6%	• Continuation of VKA (OR 12.18)<br>• Continuation of DOAC (OR 42.54)<br>• Continuation of DAPT with ticagrelor (OR 18.56)

Abbreviations: NSD=no significant differences; PPM=permanent pacemaker; ICD=implantable cardioverter-defibrillator; CRT=cardiac resynchronization therapy ; AP=antiplatelet; DAPT=dual antiplatelet therapy; SAPT=single antiplatelet therapy; ASA=aspirin; AC=anticoagulant; OAC=oral anticoagulant; VKA=Vitamin K Antagonists; HBS=heparin-bridging strategy; VKA=vitamin K antagonist; DOACs=non-vitamin K oral anticoagulants; LVEF=left ventricular ejection fraction.

Table S2. Bleeding complication rate by AP and/or anticoagulation strategy during the perioperative period

Author	Year	Case (n)	AP Therapy				AC Therapy					AP+AC (%)	No AP or AC (%)
			DAPT (%)	SAPT (%)	interrupted AP (%)	No AP (%)	heparin (%)	Continued VKA (%)	interrupted VKA (%)	DOAC (%)	No AC (%)		
Goldstein et al[86]	1998	150	N/A	N/A	N/A	N/A	N/A	2/37 (5.4)	N/A	N/A	2/113 (1.8)	N/A	N/A
Michaud et al[23]	2000	192	N/A	N/A	N/A	N/A	10/49 (20.4)	1/28 (3.6)	N/A	N/A	2/115 (1.7)	N/A	N/A
Al-Khadra et al[22]	2003	47	N/A	N/A	N/A	N/A	N/A	1/47 (2.1)	N/A	N/A	N/A	N/A	N/A
Giudici et al[87]	2004	1025	N/A	N/A	N/A	N/A	N/A	9/470 (1.9)	9/555 (1.6)	N/A	N/A	N/A	N/A
Wiegand et al[9] [a]	2004	3164	5/23 (21.7)	ASA 40/1275 (3.1) Thienopyridine 2/30 (6.7)	N/A	N/A	64/551 (11.6)	N/A	14/482 (2.9)	N/A	N/A	ASA + phenprocoumon 3/30 (10)	19/765 (2.5)
Marquie et al[68] *	2006	178	N/A	N/A	N/A	N/A	14/89 (15.7)	N/A	N/A	N/A	1/89 (1.1)	N/A	N/A
Robinson et al[21]	2009	148	N/A	N/A	N/A	N/A	Pre/post 15/67 (22) No pre/ post 2/7 (29) Pre/no post 3/39 (8)	N/A	N/A	N/A	3/35 (9)	N/A	N/A
Tischenko et al[54]	2009	272	N/A	N/A	N/A	N/A	9/38 (23.7)	9/117 (7.7)	N/A	N/A	5/117 (4.3)	N/A	N/A
Tolosana et al[2]	2009	101	N/A	N/A	N/A	N/A	4/51 (7.8)	4/50 (8.0)	N/A	N/A	N/A	N/A	N/A
Ahmed et al[89]	2010	459	N/A	N/A	N/A	N/A	7/123 (5.7)	1/222 (0.45)	2/114 (1.75)	N/A	N/A	N/A	N/A
Chow et al[24]	2010	506	N/A	N/A	N/A	N/A	21/32 (65.6)	0/46 (0)	N/A	N/A	4/428 (0.9)	N/A	N/A
Dreger et al[69] [b]	2010	427	1/109 (0.9)	N/A	N/A	3/318 (0.9)	N/A	N/A	N/A	N/A	N/A	N/A	N/A

Ghanbari et al[88]	2010	123	N/A	N/A	N/A	N/A	N/A	6/29 (20.7)	1/20 (5.0)	N/A	3/74 (4.1)	N/A	N/A
Kutinsky et al[26]	2010	935	22/100 (22)	ASA 38/463 (8.2) Clopidogrel 3/37 (8.1)	N/A	N/A	N/A	N/A	N/A	N/A	N/A	N/A	9/164 (5.5)
Przybylaki et al[60]	2010	247	13/53 (24.5)	ASA 27/194 (16.2)	N/A	N/A	N/A	N/A	N/A	N/A	N/A	N/A	N/A
Thal et al[75]	2010	200	3/15 (20)	ASA 1/82 (1.2) Clopidogrel 0/2 (0)	N/A	N/A	N/A	1/39 (2.6)	N/A	N/A	N/A	ASA + warfarin 0/13 (0) Clopidogrel + warfarin 0/1 (0) DAPT + warfarin 2/5 (40)	0/43 (0)
Tompkins et al[65 c]	2010	1388	10/139 (7.2)	ASA 21/536 (3.9)	N/A	N/A	22/154 (14.3)	3/46 (6.5)	11/258 (4.3)	N/A	N/A	N/A	4/255 (1.6)
Cano et al[27]	2011	849	8/60 (13.3)	7/220 (3.2)	N/A	N/A	23/154 (14.9)	N/A	N/A	N/A	N/A	OAC + enoxaparin bridging + SAPT 3/32 (9.4) OAC + enoxaparin bridging + DAPT 3/8 (37.5)	9/375 (2.4)
Cheng et al[6]	2011	100	N/A	N/A	N/A	N/A	2/7 (28.6)	0/50 (0)	0/43 (0)	N/A	N/A	N/A	N/A
Li et al[85]	2011	766	N/A	N/A	N/A	N/A	4/105 (3.8)	Warfarin alone (1.4)	5/243 (2.1)	N/A	N/A	ASA + warfarin 7/125 (5.6) ASA + heparin 10/117 (8.5)	N/A
Boulé et al[67 d]	2012	202	N/A	Clopidogrel 10/101 (9.9)	N/A	3/101 (3.0)	N/A	N/A	N/A	N/A	N/A	N/A	N/A
Lee et al[93]	2012	260	0/25 (0)	ASA 0/54 (0) Clopidogrel 0/4 (0)	N/A	N/A	8/14 (57.1)	N/A	N/A	N/A	N/A	N/A	N/A
Airaksinen et al[36]	2013	213	N/A	N/A	N/A	N/A	N/A	35/106 (33.2)	43/107 (40.2)	N/A	N/A	N/A	N/A

Birnie et al[4]	2013	681	N/A	N/A	N/A	N/A	54/338 (16)	12/343 (3.5)	N/A	N/A	N/A	N/A	N/A
Chen et al[52]	2013	1093	6/37 (16.2)	ASA 14/357 (3.9) Clopidogrel 1/83 (1.2)	N/A	N/A	7/50 (14)	N/A	1/54 (1.9)	N/A	N/A	N/A	11/512 (2.1)
Jennings et al[105]	2013	48	N/A	N/A	N/A	N/A	N/A	4/67 (6.0)	N/A	0/34 (0)	N/A	VKA+AP 5/128 (3.9) DOAC+AP 1/14 (7.1)	N/A
Özcan et al[10]	2013	574	1/24 (4.2)	ASA 3/196 (1.5) Clopidogrel 2/37 (5.4)	N/A	N/A	3/48 (6.3)	N/A	12/59 (20.3)	N/A	N/A	N/A	N/A
Chen et al[79]	2014	342	3/86 (3.5)	N/A	N/A	N/A	14/85 (16.5)	N/A	N/A	N/A	N/A	N/A	2/171 (1.2)
De Sensi et al[84]	2014	35	N/A	N/A	N/A	N/A	N/A	5/35 (14.3)	N/A	N/A	N/A	N/A	N/A
Kosiuk et al[111]	2014	236	N/A	N/A	N/A	N/A	N/A	9/118 (7.6)	Dabigatran 3/118 (2.5)	N/A	N/A	N/A	N/A
Kosiuk et al[104]	2014	176	N/A	N/A	N/A	N/A	N/A	N/A	Dabigatran 2/93 (2.2) Rivaroxaban 3/83 (3.6)	N/A	N/A	N/A	N/A
Nammas et al[66 e]	2014	447	N/A	ASA 7/128 (5.5)	N/A	N/A	N/A	12/213 (5.6)	N/A	N/A	N/A	N/A	1/106 (0.9)
Schulman et al[97]	2014	171	N/A	N/A	N/A	N/A	4/85 (4.7)	3/86 (3.5)	N/A	N/A	N/A	N/A	N/A
Cano et al[90]	2015	278	N/A	N/A	N/A	N/A	N/A	7/248 (2.8)	N/A	N/A	N/A	OAC + DAPT 0/1 (0) OAC + ASA 1/26 (3.8) OAC + Clopidogrel 0/3 (0)	N/A
Dai et al[12]	2015	364	6/31 (19.3)	ASA 4/124 (3.2)	N/A	4/209 (1.9)	N/A	N/A	N/A	N/A	N/A	N/A	N/A

Melton et al[3]	2015	380	N/A	N/A	N/A	N/A	N/A	9/109 (8.3)	Dabigatran 3/14 (21.4) Rivaroxaba 5/9 (55.6)	N/A	19/248 (7.7)	N/A	N/A
Amara et al[81]	2016	20	1/20 (5)	N/A	N/A	N/A	N/A	N/A	N/A	N/A	N/A	N/A	N/A
Deharo et al[38]	2016	723	9/68 (13.2)	N/A	N/A	N/A	N/A	VKA 24/210 (11.4)	N/A	1/71 (1.4)	N/A	VKA+AP 7/76 (9.2) DOAC+AP 2/26 (7.7)	N/A
Madan et al[112]	2016	133	N/A	N/A	N/A	N/A	N/A	6/86 (7.0)	Dabigatran 0/47 (0)	N/A	N/A	N/A	N/A
Tolat et al[82]	2016	748	N/A	N/A	N/A	N/A	N/A	8/230 (3.5)	N/A	N/A	2/518 (0.4)	N/A	N/A
Demir et al[53]	2017	232	4/29 (13.8)	ASA 0/73 (0) Clopidogrel 1/12 (8.3)	N/A	N/A	N/A	0/34 (0)	N/A	N/A	N/A	Warfarin + ASA 1/21 (4.8)	0/63 (0)
Malagu et al[96]	2017	1035	N/A	N/A	N/A	N/A	42/522 (6.5)	34/513 (1.6)	N/A	N/A	N/A	N/A	N/A
Ishibashi et al[130]	2017	300	0/10 (0)	2/49 (4.1)	N/A	N/A	N/A	6/89 (6.7)	N/A	N/A	N/A	OAT+SAPT 5/20 (25.0) TAT 0/3 (0)	4/129 (3.1)
Afzal et al[83]	2018	137	N/A	N/A	N/A	N/A	N/A	6/24 (25)	2/113 (1.5)	N/A	N/A	N/A	N/A
Birnie et al[16]	2018	662	N/A	N/A	N/A	N/A	N/A	N/A	N/A	7/328 (2.1)	7/334 (2.1)	N/A	N/A
Ferretto et al[34]	2020	500	3/50 (5.8)	7/179 (0.6)	N/A	N/A	11/100 (11.0)	1/31 (3.2)	N/A	1/24 (4.2)	6/254 (2.4)	AC+SAPT 2/57 (3.5) Triple therapy 3/12 (25)	1/87 (1.1)
Notaristefano et al[57]	2020	850	10/55 (18.2)	19/257 (7.4)	N/A	N/A	10/54 (18.5)	7/68 (10.3)	N/A	8/176 (4.5)	N/A	Triple therapy 5/24 (20.8)	2/208 (1.0)
Fensman et al[58]	2022	5918	31/492 (6.3)	75/2700 (2.8)	N/A	42/2726 (1.5)	10/179 (5.6)	N/A	N/A	N/A	138/5739 (2.4)	N/A	N/A
Tarakji, et al[77]	2022	6800	N/A	N/A	N/A	N/A	N/A	28/1067 (2.6)	N/A	6/455 (1.3)	N/A	57/1249 (4.6)	8/972 (0.8)

Creta, A. et al[110]	2024	1975	N/A	N/A	N/A	N/A	N/A	15/287 (5.2)	N/A	28/287 (9.8)	18/287 (6.3)	N/A	N/A

Abbreviations: Obs.=observational; RCT=randomized controlled trial; AP=antiplatelet; DAPT=dual antiplatelet therapy; SAPT=single antiplatelet therapy; HBS=heparin-bridging strategy; AC=anticoagulant; VKA=vitamin K antagonist.

* The end point was hemorrhagic and procedure-related AEs, including hematomas.

* The use of AP drug was not disclosed in some anticoagulation studies.

Note[a]: In this research, low-dose heparinization was given for perioperative prophylaxis of deep venous thrombosis in patients with a low or intermediate risk for thromboembolism (including those taking AP therapy). Patients who had received low-dose heparinization and did not received AP therapy were used as a reference group. Thus, low-dose heparinization was not counted into “anticoagulant”. Besides, the “heparin-bridging” here referred to replacing phenprocoumon by high-dose heparinization.

Note[b]: The control group consisted of 318 patients, of which 104 patients (32.7%) were on ASA and the remaining 214 control patients received no anticoagulant therapy at all. But the result didn’t describe the pocket hematoma rate in the two subgroups.

Note[c]: In the study of Tompkins et al, patients taking aspirin with warfarin or heparin were grouped together with those taking warfarin or heparin alone for purpose of analysis. The patients taking warfarin (INR >1.5) were regarded as the group “Continued VKA Therapy”, and those taking warfarin (INR <1.5) were assigned into the group “VKA Cessation”.

Note[d]: This study focused on the effect of clopidogrel on the bleeding complications after CIED implantation. Although both the clopidogrel group and control group of the study contained aspirin treatment, the specific number of patients taking aspirin was not provided. The multivariate analysis reported aspirin treatment was not independent predictors of significant bleeding complications.

Note[e]: There were 12 patients suffering significant pocket hematoma in the OAC group, which comprised 213 patients and was randomized on a 1:1 basis into two subgroups: continued OAC group, and OAC discontinued 2 days before the CRMD implantation with no heparin bridging. The effect of clopidogrel was not taken into consideration due to the limited number in this study.

Table S3. Major Studies on the Prevention Measure of Pocket Hematoma

Author	Year	Subjects	Measure		Control	Endpoint	Results
Milic et al[137]	2005	Pacemaker patients on anticoagulants	Medication	Fibrin sealant	Standard care (no fibrin sealant)	Pocket-related complications (especially hematoma)	0% hematoma in treatment group vs 14.63% in control group
Ohlow et al[141]	2012	Patients on anticoagulation/DAPT undergoing cardiac device implantation	Medication	D-Stat Hemostat™ (collagen-thrombin mixture) (n=82)	Standard care (n=81)	Clinically significant hematomas requiring evacuation	D-Stat increased hematoma evacuation rate (8.5% vs 0%)
Chia et al[134]	2013	High-bleeding-risk patients on anticoagulants/DAPT undergoing CIED implantation	Medication	Surgicel® Fibrillar™ (oxidized regenerated cellulose) (n=42)	N/A	A pocket hematoma that protrudes >2 cm (minor hematoma) or >4 cm (significant hematoma)	No PH or infection

Kiuchi et al[150]	2015	Anticoagulated patients undergoing CIED implantation	Compression	Novel compression tool (n=23)	Conventional pressure dressing (n=23)	Hematoma incidence	Hematomas 0% versus 8.7%
Ilov et al[140]	2016	Patients for primary pacemaker implantation	Medication	Local epinephrine (n=75)	Saline solution (n=58)	Pocket hematoma	Higher PH risk in epinephrine group (OR = 5)
Beton et al[138]	2016	Patients receiving antithrombotic therapy during cardiac device implantation	Medication	Topical tranexamic acid (TXA)	No TXA	Pocket hematoma and major bleeding events	3.4-3.5 fold reduction in PH and major bleeding with TXA
Koh et al[48]	2017	Patients for CIED implantation	Compression	Prophylactic pressure bandaging	N/A	Cardiac device hematoma	No significant effects on hematoma development
Tscholl et al[136]	2017	patients with high bleeding risk	Medication	PerClot® (Polysaccharide Hemostatic System) (n=25)	No PerClot (n=26)	incidence of pocket hematoma pocket infection	No significant hematoma difference; terminated early due to fever/inflammatory markers
Turagam et al[145]	2017	Patients undergoing device implantation with uninterrupted AP/anticoagulants	Compression	Post-surgical vest (n-20)	Standard care (n=20)	Pocket hematoma incidence at 7 days	0% vs 30% (p=0.02)
Mukherjee et al[142]	2018	Patients undergoing CIED implantations on APs/anticoagulants	Drainage	Closed suction drain (n=67)	N/A	Pocket hematoma incidence, other bleeding complications and the infection post procedure	Significant reduction in postoperative pocket hematoma
Awada et al[17]	2019	Patients for first CIED implantation receiving anticoagulation and/or DAPT	Drainage	Vacuum drainage system (n=103)	No exclusive measures	A composite of hematoma grade > 1 and pocket infection	No significant difference
			Medication	Stypro® (hemostatic gelatin sponge) (n=99)			HR 0.38, (0.16–0.94), p<0.05
			Compression	Premofix® (compression device) (n=103)			HR 0.37, (0.15–0.90), p<0.05 Especially hematoma grade 2 or 3
Chien et al[151]	2019	CIED recipients	Compression	Non-taped compression dress (n=85)	Conventional gauze ball and elastic tapes (n=90)	Skin integrity and the presence of oozing, hematoma, and subcutaneous emphysema around the CIED pocket	Less unfavorable outcomes in terms of skin integrity and hemostasis

Fei et al[148]	2022	Participants on uninterrupted DOACs and underwent CIED implantation	Compression	Novel compression device (n = 102)	Elastic adhesive tape with a sandbag (n = 102)	Pocket hematoma, skin erosions and patient comfort score	Significant reduction in all hematoma grades (11.8% vs 36.3%)
Hu et al[152]	2022	CIED implantation	Compression	Pocket compression device (n=54)	Elastic adhesive tape (n=54)	Pocket hematoma incidence, adverse skin reactions, the need for sandbag positional adjustments, and patient comfort	Significant reduction in hematomas (13.0% vs 44.4%) and improved comfort
Monaco et al[139]	2022	Patients underwent implantation or replacement of CIED	Medication	Gentamycin-containing collagen implant (GCCI) (n=475)	No GCCI (n=714)	Incidence of pocket hematoma and infection	Lower pocket hematoma rate (0.6% vs. 13.8%) and infection (0.2% vs. 2.6%) in GCCI group
Rojas et al[149]	2022	Patients for de novo CIED or generator change procedures	Compression	PressRite (n=51)	Standard care (n=54)	Pocket hematoma incidence, pressure measurements, standardized scar scales and tolerability	Lower pocket hematoma rate (5.9% vs. 14.8%), lower durometer readings and lower MSS score
Wei et al[143]	2022	CIED implantation	Drainage	Sub-pocket small-hole drainage (n=11)	N/A	Pocket healing, Pocket-related complications	All pockets of the 11 patients healed well without any complications
Cano et al[147]	2024	High-bleeding-risk CIED patients	Compression	Hypothermic compression bandages (n=153)	Conventional compression bandages (n=149)	Pocket hematoma prevention	Equally effective, no severe hematomas in either group
Laish-Farkash et al[131]	2025	CIED implantation with uninterrupted ATT	Medication	WALANT technique (n=24)	Standard approach (n=22)	Intraoperative hemostatic use, postoperative compression needs	Reduced hemostatic use and postoperative hematoma area
Tijskens et al[144]	2025	High-bleeding-risk CIED implantation patients	Compression	Novel adjustable transparent pocket compression device (n=32)	Elastic adhesive tape (n=33)	Pocket hematoma incidence and postoperative pain	Significant reduction in PH and postoperative pain
Prasertwitayakij et al[146]	2025	CIED implantation patients	Compression	Novel compression vest with pressure cuff (n=120)	Conventional sandbag compression (n=122)	Pocket hematoma incidence at 24h	No significant difference (26.7% vs 19.7%)

Table S4. PRISMA 2020 checklist

Section and Topic	Item #	Checklist item	Location where item is reported
**TITLE**			
Title	1	Identify the report as a systematic review.	Title
**ABSTRACT**			
Abstract	2	See the PRISMA 2020 for Abstracts checklist.	Abstract
**INTRODUCTION**			
Rationale	3	Describe the rationale for the review in the context of existing knowledge.	1. Introduction
Objectives	4	Provide an explicit statement of the objective(s) or question(s) the review addresses.	Abstract
**METHODS**			
Eligibility criteria	5	Specify the inclusion and exclusion criteria for the review and how studies were grouped for the syntheses.	2.2. Eligibility Criteria
Information sources	6	Specify all databases, registers, websites, organisations, reference lists and other sources searched or consulted to identify studies. Specify the date when each source was last searched or consulted.	2.1. Search Strategy
Search strategy	7	Present the full search strategies for all databases, registers and websites, including any filters and limits used.	2.1. Search Strategy
Selection process	8	Specify the methods used to decide whether a study met the inclusion criteria of the review, including how many reviewers screened each record and each report retrieved, whether they worked independently, and if applicable, details of automation tools used in the process.	2.3. Study Selection and Data Extraction and Figure 1
Data collection process	9	Specify the methods used to collect data from reports, including how many reviewers collected data from each report, whether they worked independently, any processes for obtaining or confirming data from study investigators, and if applicable, details of automation tools used in the process.	2.3. Study Selection and Data Extraction
Data items	10a	List and define all outcomes for which data were sought. Specify whether all results that were compatible with each outcome domain in each study were sought (e.g. for all measures, time points, analyses), and if not, the methods used to decide which results to collect.	2.3. Study Selection and Data Extraction
	10b	List and define all other variables for which data were sought (e.g. participant and intervention characteristics, funding sources). Describe any assumptions made about any missing or unclear information.	2.3. Study Selection and Data Extraction and Table S1
Study risk of bias assessment	11	Specify the methods used to assess risk of bias in the included studies, including details of the tool(s) used, how many reviewers assessed each study and whether they worked independently, and if applicable, details of automation tools used in the process.	2.4. Quality Assessment
Effect measures	12	Specify for each outcome the effect measure(s) (e.g. risk ratio, mean difference) used in the synthesis or presentation of results.	Table S1
Synthesis methods	13a	Describe the processes used to decide which studies were eligible for each synthesis (e.g. tabulating the study intervention characteristics and comparing against the planned groups for each synthesis (item #5)).	2.3. Study Selection and Data Extraction
	13b	Describe any methods required to prepare the data for presentation or synthesis, such as handling of missing summary statistics, or data conversions.	2.3. Study Selection and Data Extraction
	13c	Describe any methods used to tabulate or visually display results of individual studies and syntheses.	2.3. Study Selection and Data Extraction and Table S1
	13d	Describe any methods used to synthesize results and provide a rationale for the choice(s). If meta-analysis was performed, describe the model(s), method(s) to identify the presence and extent of statistical heterogeneity, and software package(s) used.	2.3. Study Selection and Data Extraction
	13e	Describe any methods used to explore possible causes of heterogeneity among study results (e.g. subgroup analysis, meta-regression).	Not applicable
	13f	Describe any sensitivity analyses conducted to assess robustness of the synthesized results.	Not applicable
Reporting bias assessment	14	Describe any methods used to assess risk of bias due to missing results in a synthesis (arising from reporting biases).	Not applicable

Section and Topic	Item #	Checklist item	Location where item is reported
Certainty assessment	15	Describe any methods used to assess certainty (or confidence) in the body of evidence for an outcome.	Not reported
**RESULTS**			
Study selection	16a	Describe the results of the search and selection process, from the number of records identified in the search to the number of studies included in the review, ideally using a flow diagram.	Fig 1
	16b	Cite studies that might appear to meet the inclusion criteria, but which were excluded, and explain why they were excluded.	Fig 1
Study characteristics	17	Cite each included study and present its characteristics.	3. Risk Factors of Pocket Hematoma & 4. Prevention and Treatment of Pocket Hematoma
Risk of bias in studies	18	Present assessments of risk of bias for each included study.	Not reported
Results of individual studies	19	For all outcomes, present, for each study: (a) summary statistics for each group (where appropriate) and (b) an effect estimate and its precision (e.g. confidence/credible interval), ideally using structured tables or plots.	Table S1
Results of syntheses	20a	For each synthesis, briefly summarise the characteristics and risk of bias among contributing studies.	Table S1
	20b	Present results of all statistical syntheses conducted. If meta-analysis was done, present for each the summary estimate and its precision (e.g. confidence/credible interval) and measures of statistical heterogeneity. If comparing groups, describe the direction of the effect.	Table S1~3
	20c	Present results of all investigations of possible causes of heterogeneity among study results.	Not applicable
	20d	Present results of all sensitivity analyses conducted to assess the robustness of the synthesized results.	Not applicable
Reporting biases	21	Present assessments of risk of bias due to missing results (arising from reporting biases) for each synthesis assessed.	Not reported
Certainty of evidence	22	Present assessments of certainty (or confidence) in the body of evidence for each outcome assessed.	Not reported
**DISCUSSION**			
Discussion	23a	Provide a general interpretation of the results in the context of other evidence.	5. Main Debate and Limitation & 6. Conclusions
	23b	Discuss any limitations of the evidence included in the review.	5. Main Debate and Limitation
	23c	Discuss any limitations of the review processes used.	5. Main Debate and Limitation
	23d	Discuss implications of the results for practice, policy, and future research.	5. Main Debate and Limitation
**OTHER INFORMATION**			
Registration and protocol	24a	Provide registration information for the review, including register name and registration number, or state that the review was not registered.	2 Methods
	24b	Indicate where the review protocol can be accessed, or state that a protocol was not prepared.	2 Methods
	24c	Describe and explain any amendments to information provided at registration or in the protocol.	Not applicable
Support	25	Describe sources of financial or non-financial support for the review, and the role of the funders or sponsors in the review.	Article Information and PROSPERO record
Competing interests	26	Declare any competing interests of review authors.	Article Information and PROSPERO record
Availability of data, code and other materials	27	Report which of the following are publicly available and where they can be found: template data collection forms; data extracted from included studies; data used for all analyses; analytic code; any other materials used in the review.	Not reported

From: Page MJ, McKenzie JE, Bossuyt PM, Boutron I, Hoffmann TC, Mulrow CD, et al. The PRISMA 2020 statement: an updated guideline for reporting systematic reviews. BMJ 2021;372:n71. doi: 10.1136/bmj.n71. This work is licensed under CC BY 4.0. To view a copy of this license, visit https://creativecommons.org/licenses/by/4.0/

Table S5. Abbreviation

Abbreviation	Full meaning
AC	Anticoagulant
AP	Antiplatelet
BMI	Body mass index
CIED	Cardiac implantable electronic device
CKD	Chronic kidney disease
CRT	Cardiac resynchronization therapy
CSH	Clinically significant hematoma
ICD	Implantable cardiac defibrillator
INR	International normalized ratio
PPM	Permanent pacemaker
95% CI	95% confidence interval
OR	Odds ratio
